# *Lhx5* controls mamillary differentiation in the developing hypothalamus of the mouse

**DOI:** 10.3389/fnana.2015.00113

**Published:** 2015-08-14

**Authors:** Michael Heide, Yuanfeng Zhang, Xunlei Zhou, Tianyu Zhao, Amaya Miquelajáuregui, Alfredo Varela-Echavarría, Gonzalo Alvarez-Bolado

**Affiliations:** ^1^Institute of Anatomy and Cell Biology, University of HeidelbergHeidelberg, Germany; ^2^Key Laboratory of Oral Disease and Biomedical Sciences, Stomatological Hospital, Chongqing Medical UniversityChongqing, China; ^3^Departamento de Neurobiología del Desarrollo y Neurofisiología, Instituto de Neurobiología, Universidad Nacional Autónoma de MéxicoQuerétaro, Mexico

**Keywords:** knockout, LIM-HD, mamillary body, mouse, mutant, neuronal identity, *Sonic hedgehog*

## Abstract

Acquisition of specific neuronal identity by individual brain nuclei is a key step in brain development. However, how the mechanisms that confer neuronal identity are integrated with upstream regional specification networks is still mysterious. Expression of *Sonic hedgehog* (*Shh*), is required for hypothalamic specification and is later downregulated by *Tbx3* to allow for the differentiation of the tubero-mamillary region. In this region, the mamillary body (MBO), is a large neuronal aggregate essential for memory formation. To clarify how MBO identity is acquired after regional specification, we investigated *Lhx5*, a transcription factor with restricted MBO expression. We first generated a hypomorph allele of *Lhx5*—in homozygotes, the MBO disappears after initial specification. Intriguingly, in these mutants, *Tbx3* was downregulated and the *Shh* expression domain abnormally extended. Microarray analysis and chromatin immunoprecipitation indicated that *Lhx5* appears to be involved in *Shh* downregulation through *Tbx3* and activates several MBO-specific regulator and effector genes. Finally, by tracing the caudal hypothalamic cell lineage we show that, in the *Lhx5* mutant, at least some MBO cells are present but lack characteristic marker expression. Our work shows how the *Lhx5* locus contributes to integrate regional specification pathways with downstream acquisition of neuronal identity in the MBO.

## Introduction

The hypothalamus is a brain region with essential roles in homeostasis and behavior (see for instance, Saper and Lowell, 2014). Alterations of its complex development can lead to pathological conditions in adults (Caqueret et al., 2005). The hypothalamus is subdivided into highly differentiated regions formed by functionally and morphologically highly differentiated neuronal aggregates, the hypothalamic nuclei. The induction and specification of the hypothalamus in general has been the subject of numerous studies in a variety of animal models (reviewed in Machluf et al., 2011; Pearson and Placzek, 2013). These have identified important roles for Shh, BMP, Wnt, FGF, and Nodal signaling. Although these well-known signaling pathways have been shown to specify the hypothalamus as a region, as well as determining its dorso-ventral, antero-posterior, and latero-medial axes, how the specification of individual nuclei is regulated remains elusive.

One particularly important region is the mamillary region, including its main nucleus, the mamillary body (MBO). The MBO is a large and compact neuronal aggregate acting as a hub between hindbrain, thalamus, and hippocampus through major afferent and efferent axonal bundles. The MBO has key functions in foraging behavior as in memory formation (Vann and Aggleton, 2004; Vann, 2013). Loss of the MBO in *Foxb1* mutant mice leads to deficits in working memory (Radyushkin et al., 2005). In humans, MBO degeneration is involved in the anterograde amnesia characteristic of the Wernicke-Korsakoff syndrome (Kahn and Crosby, 1972), a serious neurological condition connected to alcohol abuse (Kopelman et al., 2009) and bariatric surgery (Koffman et al., 2006). Although analysis of mouse and zebrafish mutants has shown that transcription factors *Sim1* and *2, Foxb1*, and *Fezf2* are required for MBO development and survival (Alvarez-Bolado et al., 2000a; Marion et al., 2005; Wolf and Ryu, 2013), little is known about the genetic regulation of MBO development.

Forebrain expression of *Sonic hedgehog* (*Shh*), which encodes a secreted protein with morphogen properties, is essential for appropriate hypothalamic regional specification (Szabó et al., 2009). The hypothalamic *Shh* expression domain, however, has to be downregulated in the mamillary region in order for it to differentiate (Manning et al., 2006). The T-box (Tbx) family of transcription factor genes has essential roles in development (Naiche et al., 2005; Greulich et al., 2011; Wansleben et al., 2014). Work on zebrafish has shown that Wnt inhibition is necessary for hypothalamic differentiation (Kapsimali et al., 2004), and in chick BMP signaling leads to Wnt inhibition and subsequent upregulation of T-box gene *Tbx2*, which specifically represses *Shh* in the tuberal and mamillary regions allowing them to differentiate (Manning et al., 2006). This role of *Tbx2* is performed by *Tbx3* in the mouse (Trowe et al., 2013). What is not clear is how the downregulation of Shh translates into nuclear formation and how nucleogenesis is integrated in the regulatory networks of regional specification mechanisms.

**Figure 1:**
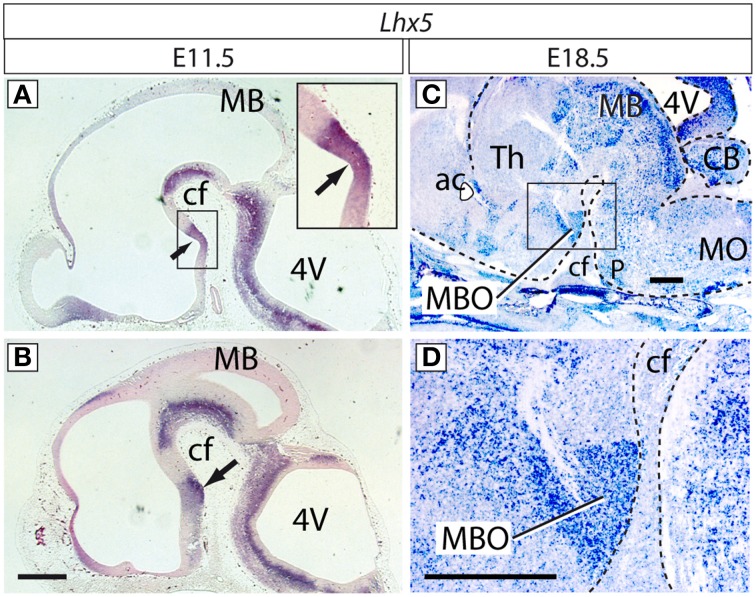
**Expression of *Lhx5* in the mamillary region**. *In situ* hybridization for *Lhx5* on sagittal sections (rostral to the left) of E11.5 (A,B) and E18.5 (C,D) brains. *Lhx5* is expressed in the ventricular zone (neuroepithelium; arrow in **(A)** and inset in **(A)** as well as in the incipient mamillary mantle layer (arrow in **B**). At E18.5, the MBO is prominently and specifically labeled in the mamillary region (framed in **C**, magnified in **D**). Abbreviations: 4V, fourth ventricle; ac, anterior commissure; cf, cephalic flexure; MB, midbrain; MBO, mamillary body; MO, medulla oblongata; P, pons; Th, thalamus. In **(C,D)** a dashed line brings out the contour of the brain. Scale bars: 500 μm.

LHX5 is a member of the LHX family of transcription factors acting as important differentiation determinants (Hobert and Westphal, 2000; Kadrmas and Beckerle, 2004), and it is strongly expressed in the caudal hypothalamus from very early stages (E9.5) through the time of formation of recognizable neuronal aggregates (Figures 1A–D) (Sheng et al., 1997; Allen-Institute-for-Brain-Science, 2009; Shimogori et al., 2010). *Lhx5* has specific roles in forebrain development— e.g., it is essential for hippocampal development (Zhao et al., 1999) and regulates the distribution of Cajal-Retzius neurons (Miquelajáuregui et al., 2010). Here we created a novel mutant allele of *Lhx5* and analyzed it using expression profiling with microarrays, ChIP-Seq and luciferase experiments, as well as examination of the hypothalamus of the *Tbx3*^−∕−^. Our results indicate that *Lhx5* has an essential role in several different developmental pathways regulating MBO specification and differentiation.

## Materials and methods

### Mouse lines

Animals were housed and handled in ways that minimize pain and discomfort, in accordance with German animal welfare regulations (Tierschutzgesetz) and in agreement with the European Communities Council Directive (2010/63/EU). The authorization for the experiments was granted by the Regierungspräsidium Karlsruhe (state authorities) and the experiments were performed under surveillance of the Animal Welfare Officer of the University of Heidelberg responsible for the Institute of Anatomy and Cell Biology. To obtain embryos, timed pregnant females of the appropriate crossings were sacrificed by cervical dislocation.

#### Conditional *Lhx5* mutant (*Lhx*5^*fl*∕+^)

**Figure 2:**
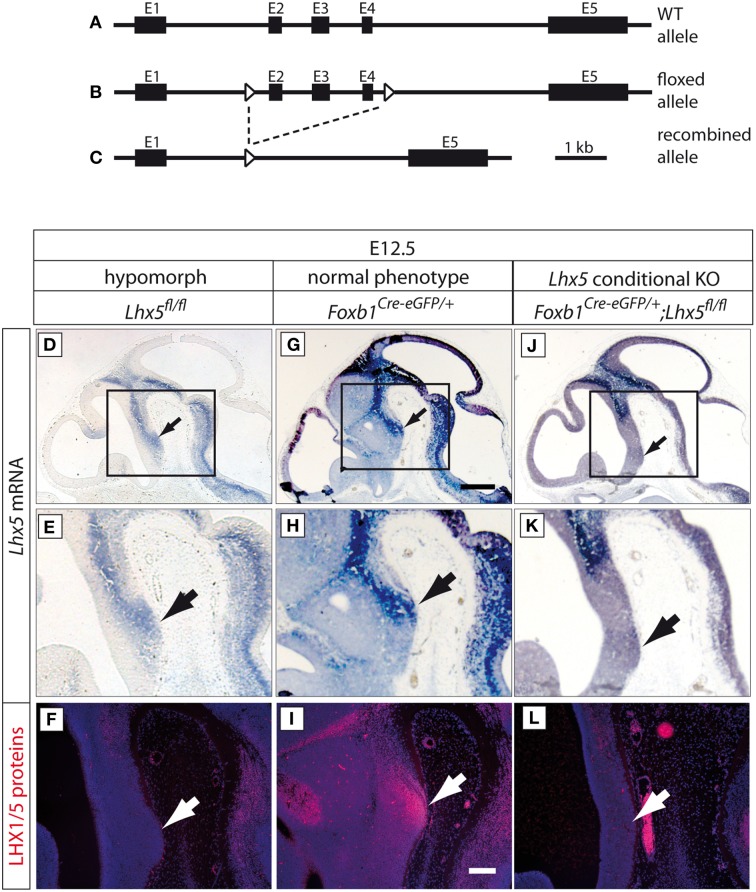
**Generation of a novel *Lhx5* mutant allele. (A–C)** *Lhx5* wild type allele, floxed allele and recombined allele, as indicated. Exons 2–4 were flanked by loxP sites. **(D–L)** Upper two rows: *In situ* detection of *Lhx5* mRNA on sagittal sections of E12.5 embryo brains (genotypes as indicated). Scale bar in **(G)**, 500 μm. Arrows show the localization of the MBO. **(E,H,K)** High-magnification of the areas framed in **(D,G,J)**. Lower row **(F,I,L)**: Antibody detection of LHX5 protein on parallel sections to those shown in **(D,G,J)**. Scale bar in **(I)**, 100 μm. Arrows show the localization of the MBO.

We generated a novel conditional allele of *Lhx5* by homologous recombination. We cloned the conditional *Lhx5* targeting construct by inserting loxP sites into the *Lhx5* locus spanning a region from intron 1 to intron 4 including exons 2–4 (Figures 2A–C).

#### *Foxb1*^*Cre−eGFP*∕+^ mutant mice

In this line (Zhao et al., 2007), the *Foxb1* coding sequence has been replaced by a Cre-IRES-eGFP cassette by homologous recombination, and this allele expresses *Cre* and eGFP under the control of the regulatory sequences of *Foxb1*. These mice show *Cre* expression in the thalamic and hypothalamic neuroepithelium (Zhao et al., 2007, 2008). We used only heterozygous *Foxb1*^*Cre*−*eGFP*∕+^ mice, which show a normal phenotype (Zhao et al., 2007, 2008), *Foxb1*^*Cre*−*eGFP*∕*Cre*−*eGFP*^ homozygotes were not used in this study.

#### ROSA26R reporter mouse line

Upon Cre-recombination, cells express ß-galactosidase (Soriano, 1999).

#### *Tbx3* mouse mutant line (*Tbx3*^+∕−^)

Histological material from *Tbx3*^−∕−^ mouse mutants (Hoogaars et al., 2007) was kindly provided by O. Trowe and A. Kispert (University of Hannover, Germany) with permission of V.M. Christoffels (University of Amsterdam, The Netherlands).

### Immunohistochemistry

Embryos or embryonic brains were dissected, fixed in 4% paraformaldehyde, embedded in paraffin, and sectioned at 10 μm. Immunofluorescent stainings were performed according to standard protocols. For antigen retrieval the sections were immersed in Tris-EDTA-Buffer pH 9 (10 mM Tris, 1 mM EDTA, 0,05% Tween-20) and treated in a Silit Sicomatic t-plus pressure cooker (2 rings, 10 min). The following primary antibodies were used: mouse anti-neurofilament 2H3 (Developmental Studies Hybridoma Bank, Iowa City, 1:100), chicken anti-GFP (Abcam Ab13970, 1:100), mouse monoclonal antibody “4F2” anti-LHX1 and LHX5 (Developmental Studies Hybridoma Bank, Iowa City, 1:100), mouse Ki67 (Becton Dickinson 550609, 1:100), mouse anti-ßIII-tubulin (Sigma-Aldrich T8660, 1:100), rat anti-BrdU (Abcam Ab6326, 1:100), mouse anti-BrdU/IddU (Becton-Dickinson 340649, 1:100), and rabbit anti-active Caspase 3 (Abcam Ab13847, 1:300). Secondary antibodies: anti-chicken IgG Alexa Fluor 488 (Life Technologies, 1:300), anti-rabbit IgG Alexa Fluor 488 (Life Technologies, 1:300), anti-mouse IgG Alexa Fluor 488 (Life Technologie, 1:300), anti-rat IgG Alexa Fluor 568 (Life Technologies, 1:300), and anti-mouse IgG Alexa Fluor 594 (Life Technologies, 1:300). Cell nuclei were stained with DAPI (Roth, 1:10000) or TO-PRO-3 (Life Technologies, 1:2000).

### Measurements of MBO size

For the measurements of MBO size (**Figure 4I**) we first visualized the MBO on sections by labeling it with an anti-GFP antibody, then measured the labeled area (in pixels) with the public domain software ImageJ. We did this for every section with MBO and added up all the MBO-section areas measured in every individual embryo. The result was the sum of the MBO section areas for mutants and controls.

### *In Situ* hybridization

Templates were amplified by PCR from cDNA and probes were synthesized using the Roche DIG RNA labeling Mix. *In situ* hybridization was performed on paraffin sections according to previously described protocols (Blaess et al., 2011). The sections were counterstained with 0.1% Eosin.

### Microarray

The mamillary neuroepithelium of E10.5 wild type and mutant embryos was dissected and directly frozen at −80°C. The RNA was preserved with RNAlater ICE (Life Technologies) and sent to MFT Services Tübingen, Germany, for microarray experiments and basic bioinformatical analysis. These experiments were done using the Affymetrix Gene Chip Mouse Gene 1.1 ST Array Plates. The “heat map” was generated by using the TM4 Software. All Microarray samples are available on the GEO database.

### Quantitative PCR

The quantitative PCR was performed according to MIQE guidelines (Bustin et al., 2009). RNA was isolated using the RNeasy Plus Micro Kit (Qiagen) and RNA integrity was checked on an agarose gel. The RNA was reverse transcribed using the M-MLV Reverse Transcriptase (Promega). Quantitative PCR was performed using Power SYBR green PCR Master Mix (Applied Biosystems) and a Step One Plus Real-Time PCR System (Applied Biosystems). Quantification was performed with the delta-delta Ct method and *Ef1* was used as endogenous control (reference gene).

### Cell culture

To generate a stably transfected *Lhx5*-expressing Neuro2a cell line, a construct was generated that added a FLAG-tag to the C-terminus of LHX5 and that controls the expression of *Lhx5* under the Ptight-promotor of the Tet-On Advanced Inducible Gene Expression System (Clontech). The cells were cotransfected with this construct and the pTet-ON-Advanced vector (3:1) by using FUGENE HD Transfection Reagent (Promega). The cells were selected in medium containing 1 mg/ml G418 and clones that showed a doxycycline-dependent, homogenous expression of *Lhx5* were frozen for further experiments.

### ChIP-Seq

The ChIP-Seq experiments were performed according to published protocols (Robertson et al., 2007; Schmidt et al., 2009).

We treated the cells of our *Lhx5*-expressing Neuro2a cells (see above) with doxycycline for 24 h to induce *Lhx5* expression, fixed the cells for 10 min in 1% formaldehyde and sonicated to generate fragments of approximately 200 bp. We performed chromatin immunoprecipitation overnight using an anti-FLAG antibody (Sigma) at 4°C, then removed protein and RNA by enzymatic digestion and sent the purified DNA to the Deep Sequencing Facility (Heidelberg University, Germany). High-throughput sequencing was performed using the NEB ChIPseq Master Mix Prep kit for Illumina and an Illumina HiSeq2000 instrument. All ChIP-Seq samples are available on the GEO database. The data obtained were analyzed using the tools of the Galaxy project (Giardine et al., 2005). Sequence reads were mapped to the mm9 genome assembly with Bowtie (Trapnell and Salzberg, 2009) and unmapped reads were removed. Peak calling was performed with MACS using a M-FOLD value of 10 and a *p*-value cutoff of 1e-05. The input sequences were used as a control. The peaks were annotated with PeakAnalyzer 1.4 (Salmon-Divon et al., 2010) using the “nearest downstream gene” method. For the identification of enriched motifs we used DREME (Bailey, 2011). As a control, we performed the same analysis with cells that only expressed the FLAG-tag. In this control only 56 peaks were found and no meaningful binding motif was identified in the DREME analysis.

### Luciferase assay

Luciferase Assays were performed in the stably transfected Neuro2a cell line using the Dual-Luciferase Reporter Assay System (Promega). The identified LHX5 binding sites were cloned into a luciferase reporter pGL4.26 (Promega) and a *Lmo1* expression vector (Origene MC203585) was used for competition experiments. TurboFect Transfection Reagent (Thermo Scientific) was used for the transfection of the *Lhx5* expressing Neuro2a cell line. Transfected cells were treated with doxycycline to induce *Lhx5* expression. Two independent experiments with triplicates were performed.

### Cell cycle analysis

The cell cycle analysis was performed according to published methods (Martynoga et al., 2005). In this protocol, timed-pregnant mice were first injected intra-peritoneally with 0.05 mg iododeoxyuridine (IddU, Sigma) in 0.9% NaCl per gram of body weight and then 1.5 h later with the same dose of bromodeoxyuridine (BrdU) (Sigma). After an additional 30 min, the mice were sacrificed and the embryos collected. Paraffin sections (8 μm) were obtained and IddU and BrdU labeled cells were detected using standard immunohistochemistry. IddU-positive and IddU/BrdU-double positive cells were quantified and the length of the S-phase and of the whole cell cycle were calculated according to published formulas (Martynoga et al., 2005).

### ß-Galactosidase activity detection

ß-Galactosidase activity was detected as described (Koenen et al., 1982). Embryos from timed pregnancies were collected and directly frozen in OCT at −80°C. The embryos were cut (20 μm) and the sections were fixed for 5 min in 1% paraformaldehyde, 0.2% glutaraldehyde and 0.2% NP-40 in PBS. The sections were then rinsed and incubated in staining solution (1 mg/ml X-gal, 5 mM K3Fe(CN)6, 5 mM K4FE(CN)6 and 2 mM MgCl2 in PBS) overnight in the dark at RT. The sections were counterstained with Nuclear Fast Red.

## Results

### *Lhx5* expression in the presumptive mamillary region

In order to investigate how the formation of the MBO is regulated we set out to analyze the function of the candidate gene *Lhx5*. *Lhx5* is expressed in the caudal hypothalamus of the mouse as early as E9.5, approximately the time when *Shh* is specifically downregulated in this area (Sheng et al., 1997) The MBO is derived from the mamillary recess (Altman and Bayer, 1986), present in the mouse from E11.5. We detected *Lhx5* transcripts by ISH at E11.5 in the presumptive mamillary neuroepithelium (ventricular zone) (medial sections) (Figure 1A) as well as in the earliest post-mitotic layer (mantle layer) of the mamillary region (Figure 1B). Later, at E18.5, when most nuclei and axonal tracts are already clearly recognizable, *Lhx5* expression persisted in the MBO labeling it in a specific and strong way (Figures 1C,D). Detailed expression patterns can be found in databases (Allen-Institute-for-Brain-Science, 2009).

The presence of *Lhx5* expression from the stage when *Shh* is downregulated in the ventricular zone until the MBO is fully formed suggested a role spanning the entire specification and differentiation of this nucleus and a possible link between the specification of the mamillary region as a separate hypothalamic field, and the specification of the MBO as a unique neuronal aggregate inside this field.

### Biallelic disruption of intronic sequences of Lhx5 causes LHX5 protein loss and abnormal phenotype

In order to explore the role of *Lhx5* in MBO development we generated a novel conditional *LHx5* mouse line (Figures 2A–C). The PGK-neo cassette was flanked by FRT sites and was removed by crossing with the FLPeR deleter mouse (Farley et al., 2000). Unexpectedly, we found high embryonic lethality for the non-Cre-recombined conditional mouse line: out of 747 mice from this line surviving after weaning only three (0.4%) were homozygous (*Lhx5*^*fl*∕*fl*^). We hypothesized that the loxP insertions in intronic regions could have disrupted a hitherto unknown regulatory element necessary for appropriate RNA processing, as has been reported in a number of other mutants (see for instance, Meyers et al., 1998; Nagy et al., 1998; Kist et al., 2005). This could result in a reduced production of LHX5 protein in *Lhx5*^*fl*∕*fl*^ mice and a hypomorph phenotype. To explore this possibility we first detected *Lhx5* mRNA on histological sections of *Lhx5*^*fl*∕*fl*^ embryo brains plus two other related genotypes as controls (Figure 2; Supplementary Figures 1, 2). As positive control we used *Foxb1*^*Cre*−*eGFP*∕+^ mouse embryos, which show normal phenotype (Zhao et al., 2007, 2008). Mouse embryos with the *Lhx5*^*fl*∕*fl*^ genotype (i.e., non-Cre-recombined) showed apparent decrease of mRNA expression as compared to *Foxb1*^*Cre*−*eGFP*∕+^ embryos (compare Figure 2D to Figure 2G; Figure 2E to Figure 2H). As negative controls we used mouse embryos with *Foxb1*^*Cre*−*eGFP*∕+^; *Lhx5*^*fl*∕*fl*^ genotypes (i.e., conditional mutants for *Lhx5*); these showed as expected loss of *Lhx5* expression in the mamillary region, where *Foxb1* and *Lhx5* normally coexpress (Figures 2J,K). Quantitation of mRNA by qPCR in *Lhx5*^*fl*∕*fl*^ and *Lhx5*^+∕+^ embryos showed a non-statistically significant tendence to smaller values in the mutant (not shown).

Then we used an antibody specific for both the LHX1 and the LHX5 proteins on parallel sections (Figures 2F,I,L) to those hybridized for mRNA. On the sections from *Lhx5*^*fl*∕*fl*^ embryos we could not detect any LHX1/5; the *Foxb1*^*Cre*−*eGFP*∕+^ embryos, on the contrary, showed strong protein expression in the mamillary body as expected (compare Figure 2F to Figure 2I). Finally, the mamillary region of the *Foxb1*^*Cre*−*eGFP*∕+^; *Lhx5*^*fl*∕*fl*^ embryos did not show LHX1/5 protein either (compare Figure 2I to Figure 2L).

**Figure 3:**
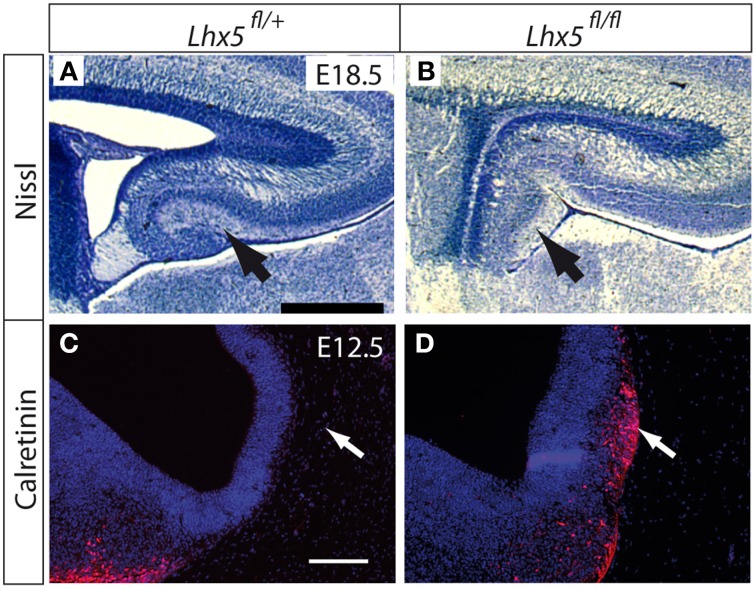
***Lhx5* mRNA and LHX5 protein in *Lhx5*^*fl*∕*fl*^ mutants**. **(A,B)** Nissl staining of the hippocampus of *Lhx5*^*fl*∕+^ **(A)** and *Lhx5*^*fl*∕*fl*^ mutant **(B)** E18.5 embryos. Arrows indicate the dentate gyrus in **(A)** and its absence in **(B)**. **(C,D)** Immunohistochemistry against calretinin (red) on *Lhx5*^*fl*∕+^ and *Lhx5*^*fl*∕*fl*^ mutant E12.5 embryos, counterstaining with DAPI (blue). Arrows in **(C,D)** show the presence of an ectopic Cajal-Retzius cell cluster in the mutant telencephalon. Scale bars: **(A,B)**: 500 μm; **(C,D)**: 100 μm.

Moreover, phenotypical analysis of *Lhx5*^*fl*∕*fl*^ embryos prior to Cre-recombination revealed a mutant phenotype resembling the published phenotypes of the *Lhx5* full mutant— a defective hippocampus (Figures 3A,B) (Zhao et al., 1999) and ectopic Cajal Retzius cells forming a cluster in the caudal telencephalon (Figures 3C,D) (Miquelajáuregui et al., 2010).

We concluded that *Lhx5*^*fl*∕*fl*^ embryos show a hypomorph phenotype probably caused by inefficient protein synthesis after insertion of a loxP site into an intron sequence with regulatory functions.

### *Lhx5* is essential for the development of the mamillary region

**Figure 4:**
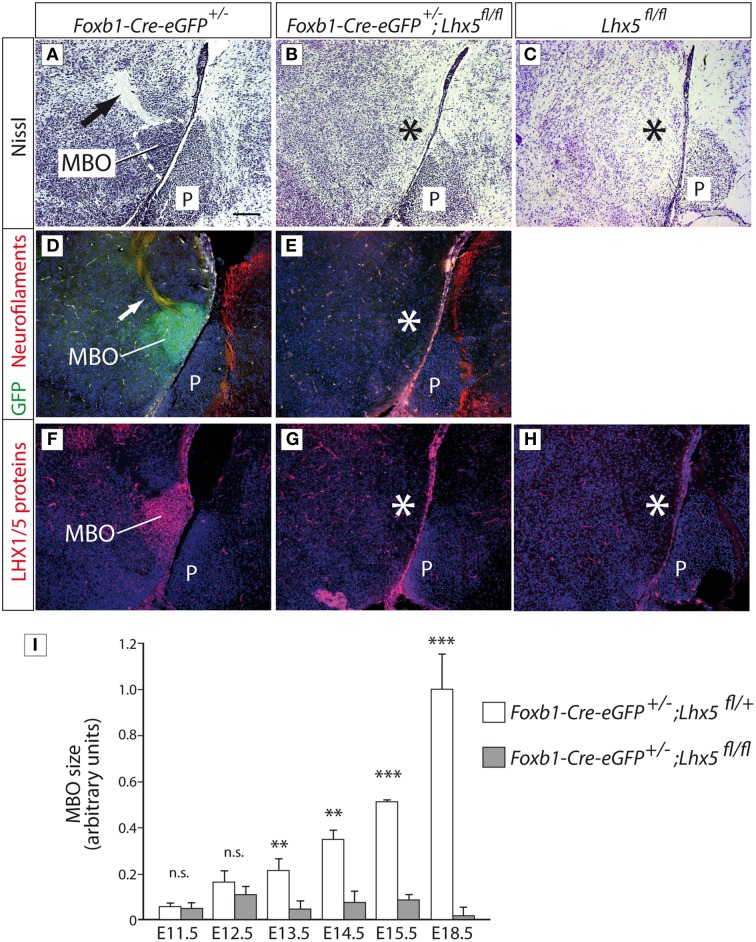
**The MBO in *Lhx5*^*fl*∕*fl*^ mutant embryos fails to increase in size**. **(A–C)** Nissl staining of sagittal sections of E18.5 brains, rostral to the left, genotypes as indicated. **(D,E)** Immunohistochemistry against GFP (green; from the reporter gene included in the *Foxb1*^*Cre*−*eGFP*∕+^ line) and neurofilaments (red), counterstained with DAPI (blue). (The *Lhx5*^*fl*∕*fl*^ mutant is not crossed with *Foxb1*^*Cre*−*eGFP*∕+^ and therefore has no eGFP reporter gene to be detected). **(F–H)** Antibody detection of LHX5 protein (red), counterstained with DAPI (blue). Arrows in **(A,D)** show the principal mamillary tract. The asterisks show the place where the MBO would be expected. Scale bar in **(A)**: 100 μm. MBO, mamillary body; P, Pons. **(I)** MBO size quantitation during development; mean ±SD; *n* = 3 embryos per age and genotype; n.s. not significant; ^*^p < 0.05; ^**^p < 0.01; ^***^p < 0.001.

To analyze the role of *Lhx5* in the development of the MBO, we crossed the *Lhx5*^*fl*∕*fl*^ conditional line with the *Foxb1*^*Cre*−*eGFP*∕+^ line (Zhao et al., 2007). Since *Foxb1* is a specific marker of the developing MBO (Alvarez-Bolado et al., 2000b), this crossing leads to a conditional inactivation of *Lhx5* in the MBO. In Nissl-stained sagittal sections at E18.5 the MBO of *Foxb1*^*Cre*−*eGFP*∕+^ mice was visible as a compact mass of neurons giving rise to a characteristic axonal bundle (the principal mamillary tract) (Figure 4A). These structures were absent in *Foxb1*^*Cre*−*eGFP*∕+^*;Lhx5*^*fl*∕*fl*^ brains (Figure 4B) as well as in *Lhx5*^*fl*∕*fl*^ brains (Figure 4C). The absence of the MBO was confirmed by loss of GFP antibody detection (enhanced GFP reporter of the iCre-IRES-eGFP-cassette), confirming the histological result (Figures 4D,E; the non-recombined *Lhx5*^*fl*∕*fl*^ brains lack of course the eGFP reporter). Finally, antibody detection of LHX5 protein (Figures 4F–H) showed that they are indeed lost in the non-recombined *Lhx5*^*fl*∕*fl*^ brains as well as in the ones from *Foxb1-Cre-eGFP*^+∕^^−^*;Lhx5*^*fl*∕*fl*^ crosses. The loss of the MBO in the non-recombined *Lhx5*^*fl*∕*fl*^ embryos was indistinguishable from that in the *Foxb1-Cre-eGFP*^+∕^^−^*;Lhx5*^*fl*∕*fl*^ embryos. To quantify the loss of the MBO we labeled sagittal sections of *Foxb1-Cre-eGFP*^+∕^^−^*;Lhx5*^*fl*∕+^and *Foxb1-Cre-eGFP*^+∕^^−^*;Lhx5*^*fl*∕*fl*^ with anti-GFP antibodies and measured the MBO area (using ImageJ software) (Figure 4I), uncovering a failure of the mutant MBO to grow to a normal size from E13.5 on.

### Proliferation and apoptosis are not altered in the *Lhx5*^*fl*∕*fl*^ MBO

**Figure 5:**
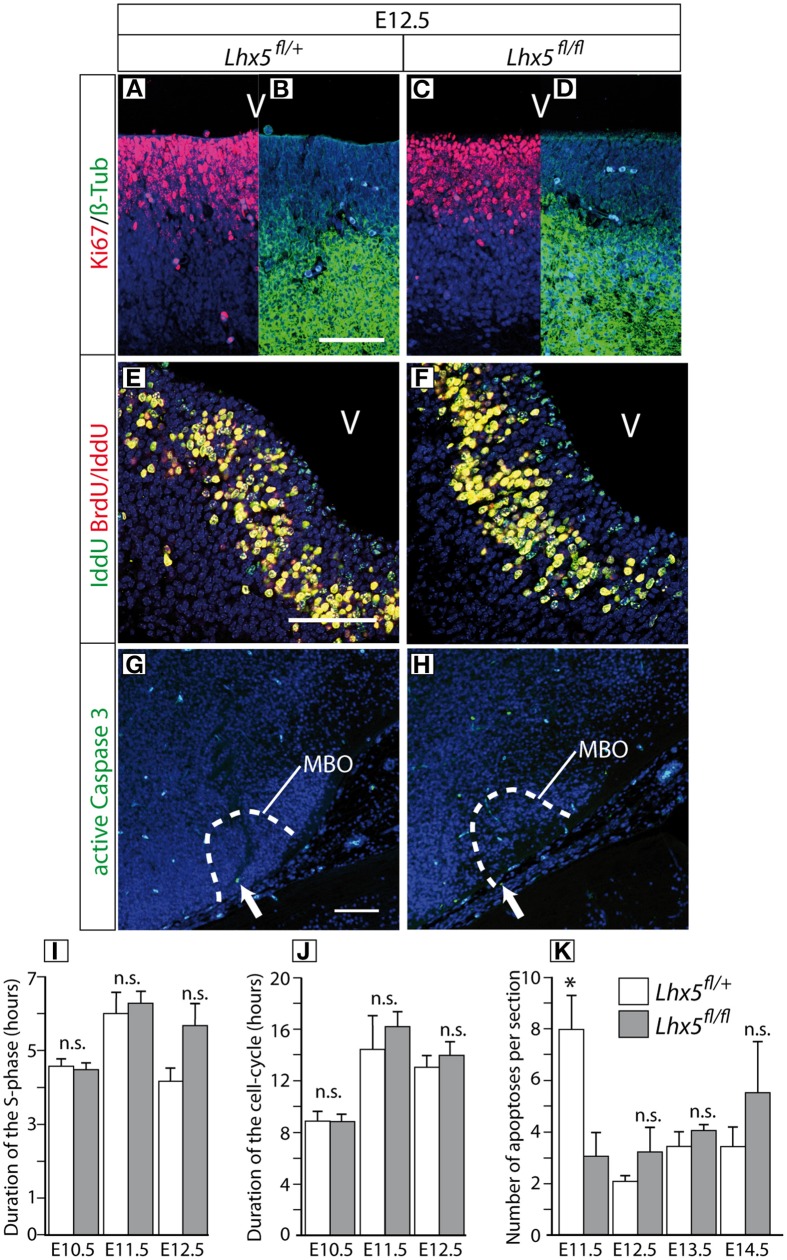
**Normal proliferation and apoptosis in the *Lhx5*^*fl*∕*fl*^ mutant**. **(A–D)** Immunohistochemistry against Ki67 (red) and ßIII-Tubulin (green) in sections through the ventricular zone of the mamillary region of E12.5 *Lhx5*^*fl*∕+^ and *Lhx5*^*fl*∕*fl*^ mutant embryos, DAPI (blue) as counterstaining. As expected, in the *Lhx5*^*fl*∕+^ **(A,B)** cells in the ventricular zone were Ki67-positive **(A)** and mantle cells (young post-mitotic neurons) were ß-III-Tubulin positive **(B)**. Similar results were found in *Lhx5*^*fl*∕*fl*^ mutant embryos **(C,D)**. **(E,F)** Immunohistochemistry to detect IddU (green) and BrdU/IddU (red) in sections of E12.5 *Lhx5*^*fl*∕+^ and mutant embryos, TO-PRO-III (blue) as counterstaining. IddU and BrdU/IddU could be detected in cells of the *Lhx5*^*fl*∕+^ as well as in cells of the mutant neuroepithelium. The signal could be clearly differentiated between Iddu positive (green) and IddU/BrdU double positive (yellow) cells **(E,F)**. **(G, H)** Immunohistochemistry for the detection of active Caspase 3 (green) in section of E12.5 *Lhx5*^*fl*∕+^ and mutant embryos, DAPI (blue) as counterstaining. **(I,J)** Neither the duration of the S-phase **(I)** nor that of the whole cell cycle **(J)** in the mamillary ventricular zone showed differences between *Lhx5*^*fl*∕+^ and *Lhx5*^*fl*∕*fl*^ mutant E10.5 through E12.5 embryos. **(K)** The number of apoptotic cells in the caudal hypothalamus was not increased in *Lhx5*^*fl*∕*fl*^ mutant E11.5 through E14.5 embryos. Abbreviations: V: ventricle, MBO: mamillary body; Arrows in **(G,H)** indicate active Caspase 3 positive cells; Scale bars: 100 μm; n.s. not significant, ^*^p < 0.05; mean ± SEM, *n* = 3 embryos.

Next we asked if either a defect in proliferation or increased apoptosis were responsible for the reduction in MBO size in the *Lhx5*-deficient hypothalamus. An initial analysis of mitotic and post-mitotic compartments in the early mamillary region using antibodies against the proliferation marker Ki67 (Starborg et al., 1996) and the neuronal marker beta-III-tubulin revealed no difference between *Lhx5*^*fl*∕+^ and *Lhx5*^*fl*∕*fl*^ (Figures 5A–D). We then applied the IddU/BrdU method (Nowakowski et al., 1989; Martynoga et al., 2005) to analyze cell cycle length in the *Lhx5*^*fl*∕+^ and *Lhx5*^*fl*∕*fl*^ mamillary neuroepithelium of E10.5–E12.5 embryos (Figures 5E,F). This method allows for calculation of the length of the S-phase of the cell cycle. One proliferation marker, IddU, is injected in pregnant mice at one time-point. After a known interval (90 min), a second proliferation marker, BrdU (which can be independently detected with specific antibodies) is injected. Both label the DNA synthesized during the S-phase. After 30 min, the embryonic brains are collected and the cells labeled either only by IddU or by both IddU + BrdU are counted. From the ratio between both numbers of cells, and since we know the interval during which cells can incorporate IddU but not BrdU (90 min.), the length of the S-phase can be easily calculated (see Martynoga et al., 2005 for details).

The *Lhx5*^*fl*∕*fl*^ showed no change in S-phase duration (Figure 5I) or in the duration of the cell cycle (Figure 5J), indicating that *Lhx5* is not essential for MBO proliferation. To investigate a possible increase in apoptosis in the *Lhx5*-deficient MBO, we labeled sections of *Lhx5*^*fl*∕*fl*^ and *Lhx5*^*fl*∕+^ caudal hypothalamus with an antibody against active Caspase3 (Figures 5G,H) and quantified the number of apoptotic cells at different developmental stages. Our results showed that the number of apoptotic cells was not increased in the *Lhx5*-deficient hypothalamus between E11.5 and E14.5 (Figure 5K). Actually, the number of apoptotic cells per section detected at E11.5 was, although small in any case, higher in control animals than in mutants (Figure 5K). Although it could be speculated about the biological significance or lack thereof of this finding, it could perhaps be a reflection of the altered properties of the mutant MBO cells already at this age.

In any case, a decrease in proliferation or an increase in cell death is unlikely causes for the reduction of the MBO in the absence of *Lhx5*.

### *Lhx5* controls MBO expression of *Lmo1* and of the cell fate determinants *Tbx3, Olig2*, and *Otp*

**Figure 6:**
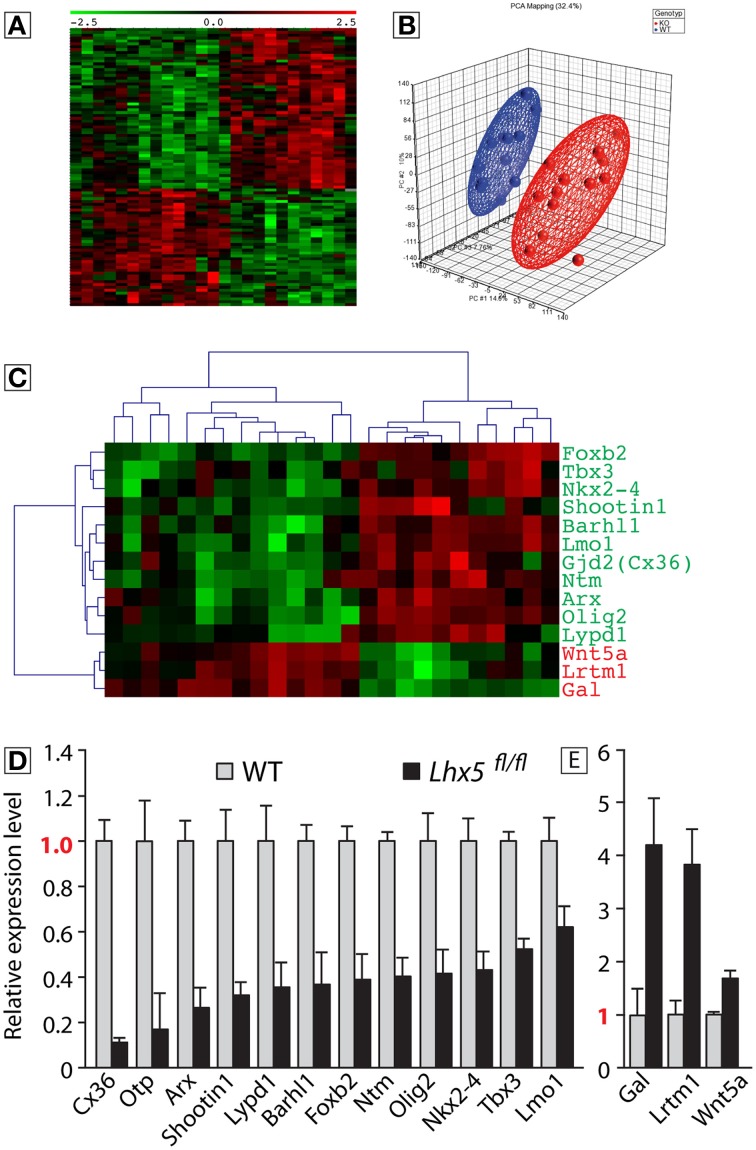
**Identification of genes that are downstream *Lhx5***. Microarray analysis of wild type and *Lhx5*^*fl*∕*fl*^ mutant E10.5 mamillary neuroepithelium and qRT-PCR validation of the identified candidates; **(A)** “Heat map” showing the expression of the identified candidates in all samples. **(B)** Principal component analysis of the microarray samples. **(C)** “Heat map” showing the expression of the candidates for qPCR analysis in all samples. **(D,E)** The qPCR validation identified 12 candidates downregulated (*p* < 0.05) **(D)** and 3 candidates upregulated in the *Lhx5*^*fl*∕*fl*^ mutant (*p* < 0.05) **(E)**. Mean ± SD; *n* = 3 biological replicates.

To understand the molecular basis of the MBO hypoplasia observed in *Lhx5*^*fl*∕*fl*^ embryos we performed comparative expression profiling using microarrays. We extracted total RNA from the caudal hypothalamus of wild type and *Lhx5*^*fl*∕*fl*^ mouse brains. Since tissue loss in the mutant could bias the results, we collected the tissue at E10.5, before any reduction in MBO size is apparent in the mutant (Figure 4I). Unsupervised hierarchical clustering of genes that were at least 1.5-fold up- or downregulated with *P* < 0.05 yielded a heat map (Figure 6A) indicating that the global gene expression patterns in embryos of the same genotype clustered together and that wild type and mutant samples were clearly distinct. Likewise, principal component analysis showed that mutant and wild type samples separate well from each other (Figure 6B). In this way we detected 56 downregulated and 41 upregulated named genes (as opposed to not yet identified transcripts like RIKEN clones, etc.) (Supplementary Table 1). After qRT-PCR validation we selected 15 candidates for further analysis (Figures 6C–E) including the cell fate determinants *Tbx3, Olig2*, and *Otp* as well as *Lmo1*, an interaction partner of LDB, the obligate cofactor of LHX proteins (Bach, 2000). In order to visualize the changes in spatial expression patterns, *in situ* hybridization analysis on tissue sections of E12.5 *Lhx5*^*fl*∕+^ and *Lhx5*^*fl*∕*fl*^ embryos was performed (Supplementary Figure 3). All candidate genes downregulated in the *Lhx5*^*fl*∕*fl*^ mutant (Figures 6C–E) were expressed in the *Lhx5*^*fl*∕+^ mamillary region (either in the neuroepithelium or in the mantle layer) and appeared reduced or absent in the mutant (Supplementary Figure 3). Some downregulated candidates showed complete loss of expression: *Foxb2* (Supplementary Figures 3E,F), *Ntm* (Supplementary Figures 3Q,R), *Lypd1* (Supplementary Figures 3S,T) and *Cx36* (Supplementary Figures 3U,V). Others showed a strong reduction in labeling: *Otp* (Supplementary Figures 3G,H), *Barhl1* (Supplementary Figures 3K,L), *Nkx2.4* (Supplementary Figures 3C,D), *Olig2* (Supplementary Figures 3I,J). Finally, other downregulated candidates showed pattern changes, like *Tbx3* (Supplementary Figures 3A,B). As for the upregulated, *Wnt5a* (Supplementary Figures 3C′,D′) and *Lrtm1* (Supplementary Figures 3W,X) showed clear ectopic expression in the mutant MBO, while *Gal* (Supplementary Figures 3A′,B′) showed increase in intensity.

The microarray data have been deposited in NCBI's Gene Expression Omnibus (Edgar et al., 2002) and are accessible through the GEO Series accession number GSE61614 and the link http://www.ncbi.nlm.nih.gov/geo/query/acc.cgi?token=yxgbokgqrvkdjif&acc=GSE61614.

### LMO1 is a possible functional antagonist of LHX5

**Figure 7:**
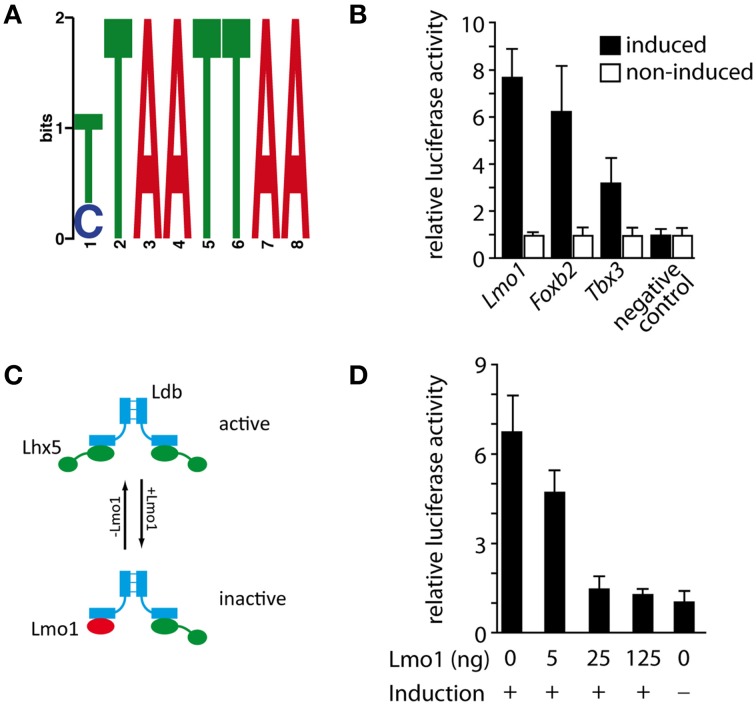
**Identification of direct LHX5 targets and negative regulation of the LHX5 function by LMO1**. **(A)** Position weight matrix of the binding motif enriched in the identified LHX5 binding sequences. **(B)** Luciferase assay validation of the identified LHX5 binding sites for *Lmo1, Foxb2*, and *Tbx3* in the doxycyclin-dependent *Lhx5*-expressing Neuro2a cell line. Mean ± SD, *p* < 0.001. **(C)** Model of the regulatory function of LMO1 on LHX5 that we propose (see the Results Section for details). **(D)** Luciferase assay in the doxycyclin-dependent *Lhx5*-expressing Neuro2a cell line stably transfected with a vector expressing luciferase under the control of the LHX5 binding site found in *Lmo1*. Increasing amounts of an *Lmo1* expression vector were cotransfected. Mean ± SD.

In order to elucidate whether the regulatory interactions observed above are direct we used chromatin immunoprecipitation followed by massively parallel sequencing (ChIP-Seq) (Robertson et al., 2007). Performing this analysis on primary tissue would have been the best choice. This was however not possible since there is no ChIP-grade available antibody against LHX5 (and the only antibody against LHX5 known to us identifies also LHX1). For this reason we chose to transfect a construct expressing a fusion protein of Lhx5 plus FLAG tag (which can be reliably identified with antibodies) into a stable cell line known to express *Lhx5*. Then we used the Tet-On Advanced Inducible Gene Expression System to regulate the *Lhx5* expression level so that it mimics the natural expression level (see Materials and Methods for details). We identified 546 possible LHX5 binding sites, which we assigned to corresponding genes using the nearest downstream method (Peak Annotation with Peak Analyzer). We then analyzed these binding sites for enriched motifs using the DREME software (Bailey, 2011) and found a motif (Figure 7A) that is enriched in 32.18% of the binding sites and corresponds to a predicted LHX5 binding motif (Berger et al., 2008). Of the loci corresponding to qPCR-validated microarray candidates, three showed this motif— *Lmo1, Tbx3*, and *Foxb2*. We then performed luciferase assays to test whether these binding sites can regulate expression of their downstream genes; the results indicated (Figure 7B) that *Lmo1, Foxb2*, and *Tbx3* are possible direct targets of LHX5 (Figure 7B). LIM-domain-only (LMO) proteins (like LMO1) can negatively regulate the function of LIM-HD transcription factors by competing with them for binding to the dimer of their obligate co-factor LIM domain-binding protein (LDB) (Bach, 2000; Chen et al., 2010). When the two binding domains of the LDB dimer are occupied by two copies of a LIM-homeodomain protein (like LHX5), this protein is active as a transcriptional regulator. On the contrary, if one of the LIM copies is substituted by an LMO protein, the LIM-homeodomain transcription factor is not active anymore. The downregulation of *Lmo1* that we observed in the *Lhx5* mutant suggests that transcription of this negative LHX regulator, in turn, is activated by LHX5, thereby providing a negative feedback loop for *Lhx5* (Figure 7C). We used luciferase assays to test this hypothesis and found that LMO1 exerts dose-dependent inhibition of transcriptional activation from the LHX5 binding site (Figure 7D). In summary, we showed that *Tbx3, Foxb2*, and *Lmo1* are possible direct targets of LHX5 and, additionally, that LMO1 negatively regulates LHX5 via a negative feedback loop.

### Tbx3 has a role in MBO development

**Figure 8:**
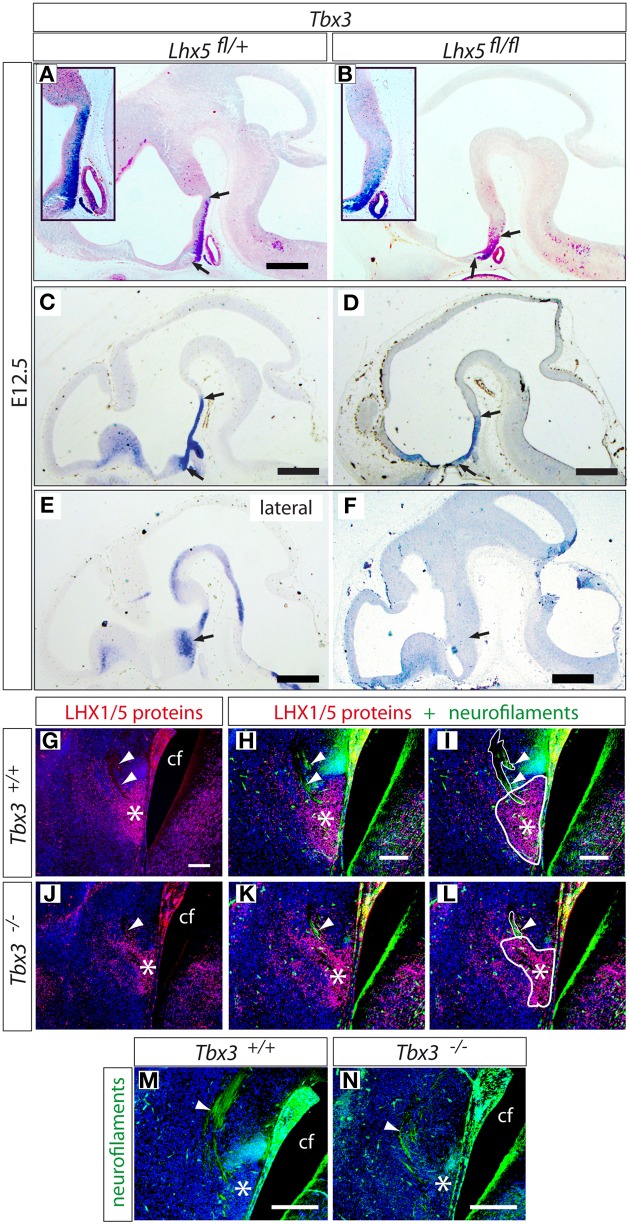
***Tbx3* expression is reduced in the *Lhx5*^*fl*∕*fl*^ mutant**. **(A–F)** *in situ* detection of *Tbx3* expression in *Lhx5*^*fl*∕+^ and *Lhx5*^*fl*∕*fl*^ mutant E12.5 embryos. Three different medio-lateral levels are shown (from top to bottom). Arrows indicate the boundaries of *Tbx3* expression in **(A–D)** and the lateral extension of the *Tbx3* expression domain in **(E,F)**. **(G–N)** Immunohistochemistry against LHX1/5 (red) and neurofilaments (green) in *Tbx3* −∕− and control E14.5 embryos, counterstaining with DAPI. **(G,J)** show detection of LHX1/5 in the MBO of control **(G)** and *Tbx3*−∕− mutant **(J)**. **(H,K)** show LHX1/5 plus neurofilaments in the MBO of control **(H)** and *Tbx3*−∕− mutant **(K)**. **(I,L)** show the same panels as in **(H,K)** with thick lines indicating the MBO profile and thin lines indicating the principal mamillary tract axons. **(M,N)** show a higher magnification view of the axons of the principal mamillary tract in controls and mutants. Arrowheads indicate the axons of the principal mamillary tract. Asterisks indicate the position of the MBO. Scale bars: **(A–F)**, 500 μm; **(E–N)**, 100 μm.

One of these genes, *Tbx3*, has an important role in the development of the hypothalamus (Manning et al., 2006; Trowe et al., 2013). We examined the expression of *Tbx3* at three different medio-lateral levels in our mutants at E12.5 (Figures 8A–F) and found a strong reduction in the expression domain in the mutant. This reduction affected not only the rostro-caudal extension of the midline (Figures 8A,C vs. Figures 8B,D) but was also evident at more lateral levels (Figures 8E,F). We then hypothesized that *Tbx3* is involved in MBO development, and on this basis predicted MBO defects in *Tbx3*-deficient brains. Examination of the hypothalamus of *Tbx3* mutant mice (Hoogaars et al., 2007) at E14.5 showed a reduced MBO with abnormal morphology (Figures 8G–L) as well as a strong reduction in axonal projections (Figures 8M,N). Since the *Tbx3* mutant embryos die before birth, usually around E14.5, we could not ascertain the possible total loss of the MBO at later stages.

### The *Shh* domain is enlarged in *Lhx5* mutant hypothalamic midline

**Figure 9:**
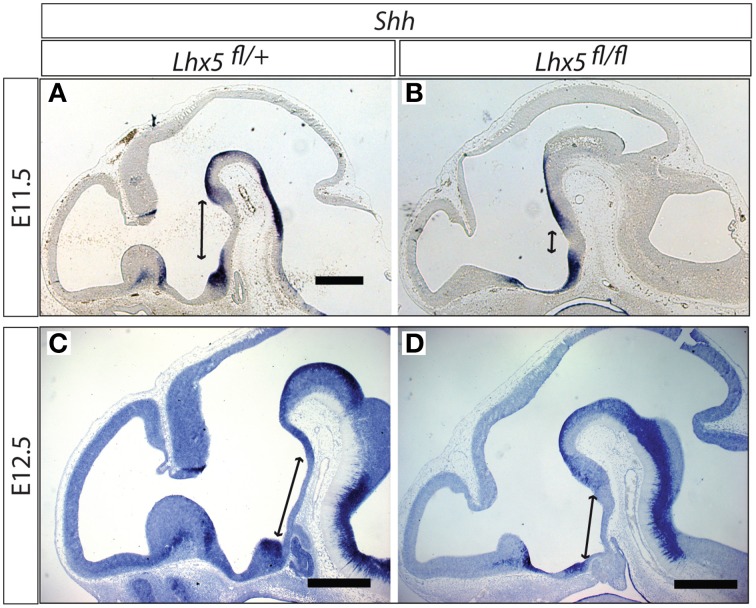
***Shh* domain expanded in the *Lhx5*^*fl*∕*fl*^ mutant**. *In situ* detection of *Shh* expression in *Lhx5*^*fl*∕+^ and *Lhx5*^*fl*∕*fl*^ mutant E11.5 **(A,B)** and E12.5 **(C,D)** embryos. Lines with arrowed ends indicate the size of the *Shh*-free region in the midline. Scale bars: 500 μm.

The reduction in *Tbx3* is very intriguing, since this gene (*Tbx2* in chicken) is needed to inhibit *Shh* expression in the tubero-mamillary region (Manning et al., 2006; Trowe et al., 2013), an event indispensable for this region to differentiate (Manning et al., 2006). We hypothesized that the downregulation of *Tbx3* in *Lhx5* mutants would result in an abnormal expansion of the territory of *Shh* in the caudal hypothalamus. *In situ* detection of *Shh* expression confirmed that, in the *Lhx5* mutants, the domain where *Shh* is normally downregulated becomes very small (Figures 9A,B). We obtained similar results at E12.5, when *Shh* expression is at its peak in this region, after which it starts to disappear (Figures 9C,D). We concluded that failure to completely inhibit Shh expression in the tubero-mamillary region is a possible mechanism explaining the MBO phenotype that we observe in the *Lhx5* mutants.

### The *Foxb1* lineage is deficiently specified in the Lhx5-Deficient caudal hypothalamus

**Figure 10:**
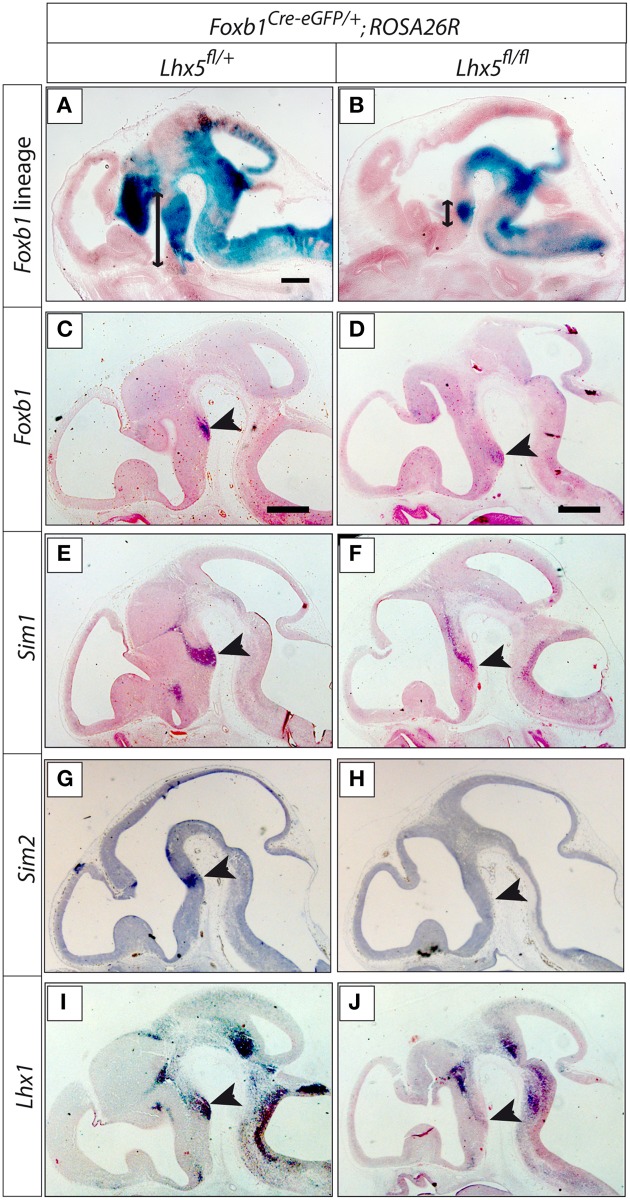
**The *Foxb1* lineage of the MBO is present in the *Lhx5*^*fl*∕*fl*^*mutant***. **(A,B)** ß-galactosidase activity (blue) reveals the *Foxb1* lineage in sagittal sections of E13.5 embryos of *Foxb1*^*Cre*−*eGFP*∕+^*;ROSA26R;Lhx5*^*fl*∕+^ **(A)** and *Foxb1*^*Cre*−*eGFP*∕+^*;ROSA26R;Lhx5*^*fl*∕*fl*^ **(B)**. Arrows show the extension of the *Foxb1* lineage in the caudal hypothalamus. **(C–J)** *In situ* hybridization detection of marker gene expression (as indicated) on sagittal sections of E12.5 *Lhx5*^*fl*∕+^ and *Lhx5*^*fl*∕*fl*^ mutant embryos. Arrowheads mark the position of the MBO. Scale bars: 500 μm.

Other transcription factor genes downregulated in our *Lhx5* mutant are *Olig2* and *Otp*, known to be involved in MBO development (see Discussion), as well as *Foxb2, Barhl1, Nkx2-4*, and *Arx*. Since we did not detect changes in apoptosis or proliferation defects, it seems likely that the cells constituting the MBO primordium are present in the mutants but that they have lost their specific MBO identity. The subsequent loss of cell adhesion protein expression (*Cx36-Gdj* and *Ntm*) could underlie the loss of morphological appearance of the MBO and make it undetectable. Since *Foxb1* is an early marker of the mamillary neuroepithelium and the developing MBO (Kaestner et al., 1996; Alvarez-Bolado et al., 2000b), we used β-galactosidase detection in *Foxb1-Cre;ROSA26R* mice to reveal the MBO lineage. In *Foxb1-Cre*^+∕^^−^*;Lhx5*^*fl*∕+^*;Rosa26R*^+^ a large caudal hypothalamic domain including the mamillary area was labeled in E13.5 embryos (Figure 10A). In *Foxb1-Cre*^+∕^^−^*;Lhx5*^*fl*∕*fl*^*;Rosa26R*^+^, however, there is only a restricted, round domain formed by cells of the *Foxb1* lineage at E13.5 (Figure 10B), corresponding in appearance and position to the MBO. We assume that these are abnormally undifferentiated cells originally fated for the MBO. Expression analysis of specific MBO markers *Foxb1, Lhx1, Sim1* and *Sim2* (Figures 10C,E,G,I) showed strong downregulation in the mutant (Figures 10D,F,H,J). Additionally, the preserved expression of *Lhx1* in regions other than the mamillary (Figures 10I,J) indicates that the result is specific. This result confirmed the presence of MBO cells with abnormal loss of identity in our mutant.

### Overall hypothalamic regional specification appears correct in the Lhx5 mutant

**Figure 11:**
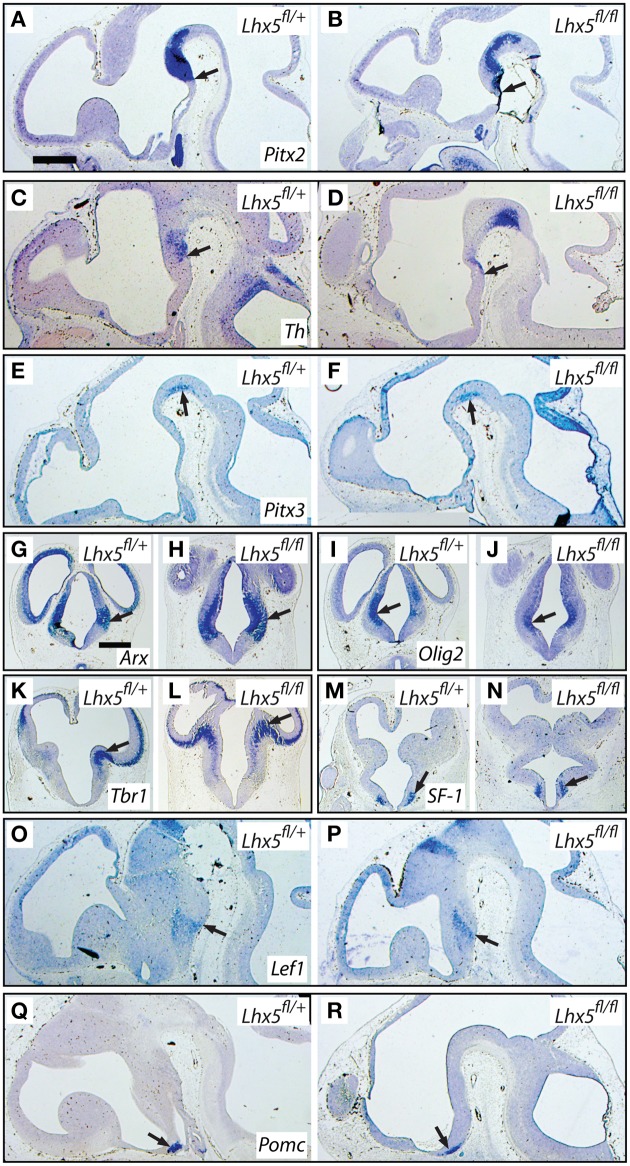
**The tuberal and prethalamic regions seem unaffected in the *Lhx5*^*fl*∕*fl*^ mutant**. *In situ* hybridization detection of marker gene expression (as indicated) on sections of E12.5 embryos of in *Lhx5*^*fl*∕+^ and *Lhx5*^*fl*∕*fl*^ mice (as indicated). Arrows indicate comparable points in the expression domains of controls and mutants. **(A–F)** *Pitx2, Th*, and *Pitx3* are midbrain markers. **(G–J)** *Arx* and *Olig2* are prethalamus markers. **(K,L)** *Tbr1* is a marker of the thalamic eminence. **(M–R)** *SF-1, Lef1* and *Pomc* are tuberal markers. See Results Section for details. Scale bars: 500 μm (in **A**, for sagittals; in **G**, for transverse sections).

To learn more about the extension of the changes observed in the *Lhx5* mutant, we performed a general analysis with markers for adjacent regions as well as markers of the tuberal region. Hypothetically, *Lhx5* might act in posterior hypothalamic progenitors to repress midbrain identity and at the same time promoting expression of MBO markers. Therefore, in the mutants, the rostral end of the ventral midbrain would abnormally extend into the mamillary region of the hypothalamus. We tested this hypothesis by detecting three genetic markers of the ventral midbrain whose expression patterns inform about the rostral extension of the ventral midbrain: *Pitx2, tyrosine hydroxylase* (*Th*), and *Pitx3* (Figures 11A–F). The domains of these three markers were essentially unaltered in the mutants (see arrows in Figures 11A–F), which belied any expansion of the midbrain domain into the *Lhx5*-deficient hypothalamus. *Arx* and *Olig2* are markers of the prethalamus, an *Lhx5*-expressing region adjacent to the hypothalamus. Expression of both genes was maintained in the mutants (Figures 11G–J). The expression of *Tbr1*, a marker of the thalamic eminence, was not affected in the mutants either (Figures 11K,L). Finally, we explored the expression of several markers of the tuberal region. The expression of genes specific for some important hypothalamic nuclei, like *SF-1* (*Nr5a1*) (marker of the ventromedial nucleus of the hypothalamus) and *Pomc* (marker of the arcuate nucleus) was not changed in the *Lhx5* mutants (Figures 11M–P). *Lef1*, a marker of the boundary between the mammillary and tuberal regions, was also essentially unchanged in the mutants (Figures 11Q,R). Additionally, we performed apoptosis (caspase 3) and proliferation (Ki67) analyses on the hypothalamic tuberal region as well as the prethalamus, which showed no change in the mutant (not shown).

We concluded that the hypomorph allele of *Lhx5* that we have generated does not cause a general defect in hypothalamus development, but its effects are mostly felt on the MBO. This said, we have also observed gross alteration of pituitary development in our mutants (not shown), due probably to the reduction in *Tbx3* expression (*Tbx3* is essential for pituitary development, Trowe et al., 2013).

## Discussion

**Figure 12:**
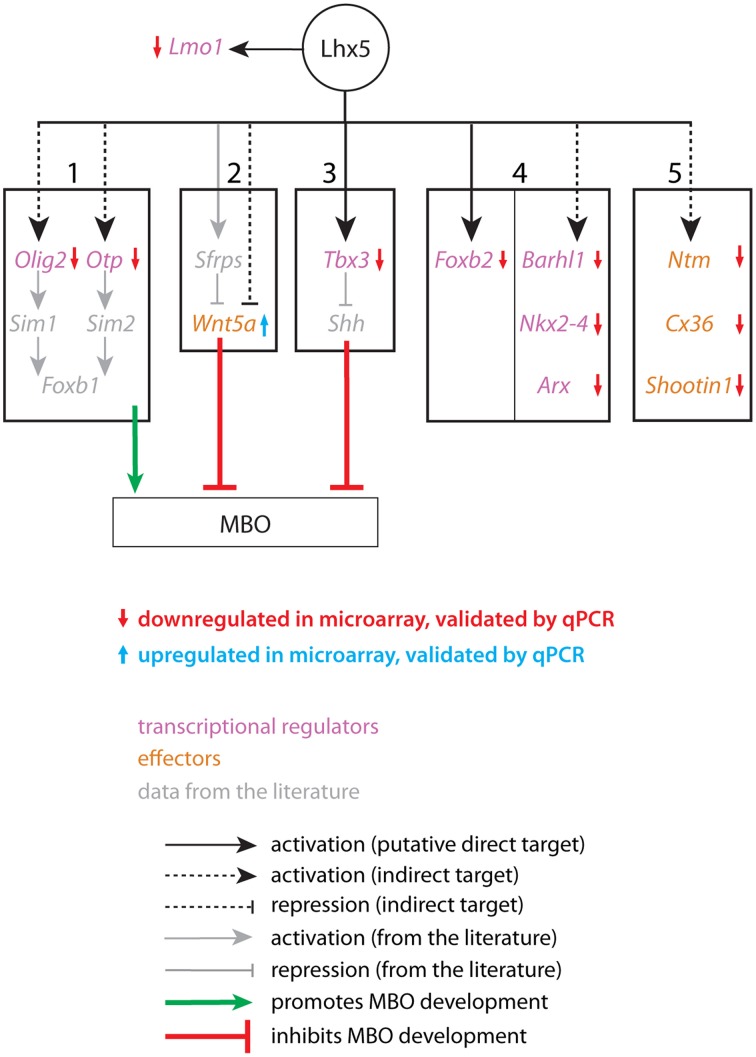
**A possible network of *Lhx5*-regulated genes and interactions related to MBO development and differentiation**. Genes downregulated (red arrows) or upregulated (blue arrows) according to microarray data validated by qPCR have been placed into 5 “bins”. Bins 1–3 contain genes and interactions known to be essential for MBO development (bin 1) or for the development of the hypothalamus (bins 2 and 3). Bin 4 shows transcription factors specifically expressed in the MBO which have not been proven essential for its development. Bin 5 contains effector genes (adhesion, axonal guidance) expressed in the MBO and presumably involved in its differentiation. *Lmo1* has a reciprocal regulatory relation with *Lhx5* not shared with any of the other candidates. In gray, data from the literature.

We attempted to elucidate the mechanisms downstream *Shh* signaling by which the regions of the caudal hypothalamus acquire their identity. The expression domain of *Lhx5* is appropriate for this gene to play a role in determining important properties of the caudal hypothalamus. Therefore, we generated a novel mutant allele giving rise to a hypomorph. Subsequent expression analysis with microarrays and other experiments have provided us with a series of candidate genes involved in appropriate differentiation of the MBO. LHX5 regulates directly or indirectly the onset or the maintenance of expression of these genes and is therefore key to MBO development (Figure 12). Two major pathways known to be involved in the development of the tubero-mamillary region could be affected in the *Lhx5* mutant. One of them includes transcription factors *Olig2* and *Otp* acting upstream of *Sim1* and *2* and finally *Foxb1* for differentiation and survival of the MBO. The second involves the restricted inhibition of *Shh* by *Tbx3* to allow for tubero-mamillary differentiation.

### *Olig2* and *Otp* act upstream of *Sim1* and *Sim2* in MBO development

Of the genes found in our expression analysis, some can be readily related to the MBO phenotype. We have divided them into five categories (Figure 12, bins 1–5). The first one contains the transcription factors *Olig2* and *Otp. Olig2* activates *Sim1* in the developing zebrafish diencephalon (Borodovsky et al., 2009), and the expression domain of *Sim1* was strongly reduced in our mutant (Figures 10E,F). *Otp* is required for *Sim2* expression in the mouse hypothalamus (Wang and Lufkin, 2000). Both *Sim1* and *Sim2* are essential for MBO development (Marion et al., 2005) and additionally they are required to maintain MBO expression of the transcription factor *Foxb1*, which is itself required for MBO development and survival (Alvarez-Bolado et al., 2000a). This suggests that *Lhx5* contributes to initiate or maintain a transcriptional network that is required for the specification of the MBO.

### *Tbx3* is required for MBO development downstream of *Lhx5*

In the chicken hypothalamus, *Tbx2* is required to antagonize Shh in the tubero-mamillary region in this way allowing it to acquire hypothalamic fate (Manning et al., 2006). The same function is performed by *Tbx3* in the mouse (Trowe et al., 2013). We show here that *Tbx3* is a possible direct target of *Lhx5* (Figure 6B), that the *Shh* expression domain is inappropriately large in our mutant (Figures 9C,D), and that the *Tbx3*-deficient brain has an abnormal MBO (Figures 8G–L). Thus, *Lhx5* is upstream of three pathways all of which can independently cause the MBO defects observed in our mutants (summarized in Figure 12, bins 1–3).

### Is *Wnt* inhibition through *Lhx5* required for MBO development?

In zebrafish (Kapsimali et al., 2004) and chicken (Manning et al., 2006) hypothalamic fate acquisition requires Wnt pathway inhibition. *Lhx5* inhibits Wnt by acting upstream of the Wnt antagonists *Sfrp1a* and *Sfrp5* in zebrafish (Peng and Westerfield, 2006). Although to the best of our knowledge these interactions have not yet been confirmed in the mouse, our observation that *Wnt5a* (Figure 12, bin 2) is ectopically expressed in the MBO (Supplementary Figures 3C′,D′) seems to agree with them. This would suggest that a lack of antagonism of Wnt signaling in this region may lead to a failure to acquire appropriate fate. On the other hand, the increase in Wnt5a that we observe is very small, and there are no signs of other genes of the Wnt pathway altered in our mutant. For this reason we consider that this result suggests some Wnt involvement in MBO development, in general agreement with published data on other models. But this line of inquiry would need to be confirmed by further experiments in the mouse.

### Deficient MBO specification in the *Lhx5* mutants

The final result of these alterations could be a reduction in the region specified to produce MBO neurons as well as an imperfect differentiation of the MBO-fated neurons that could still be generated. *Lhx5* mutants would have fewer MBO neurons and they would be incorrectly specified. This deficient specification would in turn cause loss of expression of specific markers (Figure 12, bins 4, 5). Furthermore, the genes in the fifth bin are involved in adhesion (*Ntm, Cx36-Gdj*) and axonal outgrowth (*Shootin1*), which suggests that the loss of MBO identity may additionally translate into a loss of specific aggregation of MBO neurons and loss of the characteristic mamillary axonal tree. The lack of changes in proliferation or apoptosis (Figure 5) together with the persistence of a restricted group of *Foxb1*-lineage cells in the mamillary region (Figures 10A,B) are consistent with this hypothesis.

### LMO1 is a possible direct target and antagonist of Lhx5

LMO proteins antagonize the function of LHX transcription factors by competing with them for binding to the LHX obligate partner LDB (Bach, 2000). Since we show that *Lmo1* is a possible direct target of LHX5, we predict that *Lmo1* and *Lhx5* are arranged in a negative feedback loop and our luciferase assays confirm this prediction (Figure 7D). A similar mechanism is in operation in the developing thalamus between *Lhx2* and *Lmo3* (Chatterjee et al., 2012).

### Zebrafish vs. mouse

The function of hypothalamic regulators seems to have been highly conserved during evolution and zebrafish orthologs have similar roles to their mouse counterparts (Machluf et al., 2011). Therefore, it would be interesting to know if the proposed gene regulatory network is evolutionary conserved and valid in other organisms. In zebrafish, the mamillary region is specified by the combined activity of transcription factors *Fezf2, Otp, Sim1a*, and *Foxb1.2* (Wolf and Ryu, 2013). At a stage when neuronal specification takes place, the expression domains of *Fezf2, Otp, Foxb1.2*, and *Sim1a* form distinct subdomains within the zebrafish mamillary region giving rise to distinct mamillary neuronal subpopulations (Wolf and Ryu, 2013). Here we show that in the mouse *Otp, Sim1*, and *Foxb1* are direct or indirect targets of LHX5, which is essential for MBO development. *Fezf2* is expressed early in the developing forebrain and controls regionalization of the diencephalon in both zebrafish and mouse (Hirata et al., 2006; Jeong et al., 2007; Shimizu and Hibi, 2009; Scholpp and Lumsden, 2010). Furthermore, it is expressed in the mouse mamillary neuroepithelium, but not throughout the entire MBO (Allen-Institute-for-Brain-Science, 2009). *Fezf1*- and *Fezf2*-are responsible for the expression of *Lhx5* in the subthalamus, and the double mutant mouse exhibits a hippocampal phenotype very similar to that of the *Lhx5* mutant (Zhao et al., 1999; Hirata et al., 2006). However, the MBO was intact in this double mutant (Hirata et al., 2006). These results suggest that the pathways underlying hypothalamic regional development are conserved to a high degree.

### Conflict of interest statement

The authors declare that the research was conducted in the absence of any commercial or financial relationships that could be construed as a potential conflict of interest.

## Supplementary material

Supplementary Table 1 **List of results of microarray expression profiling**.

Supplementary Figure 1 **Medio-lateral series of sagittal sections through the brain of mouse embryos (E12.5), genotypes as indicated at the top**. For each genotype, the left column shows in situ hybridization for *Lhx5* and the right column shows labeling of an adjacent section with anti-LHX1/5 antibody (at higher magnification). The arrows indicate the mamillary body primordium. Scale bars left column (ISH), 500 ìm; right column (antibodies), 100 μm.

Supplementary Figure 2 **Same as Supplementary Figure 1, for the Foxb1-Cre-eGFP^+/−^ x Lhx5^*fl/fl*^ genotype**.

Supplementary Figure 3 **Expression pattern of qPCR-validated candidates on Lhx5^*fl*/+^ and Lhx5^*fl/fl*^ mutant embryos In situ hybridization of qPCR-validated microarray candidates (as indicated) on sagittal sections of E12.5 Lhx5^*fl*/+^ and Lhx5^*fl/fl*^ mutant embryos**. Insets show MBO under higher magnification. Black arrows indicate the position of the MBO; arrows after the gene name indicate downregulation (red arrow) or upregulation (blue arrow) in microarrays and qPCR. Scale bar (in A): 500 μm.
