# Supplementary material for: Lhx5 controls mamillary differentiation in the developing hypothalamus of the mouse
Source: Front Neuroanat. 2015 Aug 14;9:113. doi: 10.3389/fnana.2015.00113 (PMC4536661; doi:10.3389/fnana.2015.00113)
Supplement: Supplementary Table 1 — List of results of microarray expression profiling. [file Table1.PDF]

| Gene Symbol | RefSeq | p-value(Genotyp) | p-value(KO vs. WT) | Ratio(KO vs. WT) | Fold-Change(KO vs. WT) | Fold-Change(KO vs. WT) (Description) |
|---|---|---|---|---|---|---|
| Gpr50 | NM_010340 | 3,39E-05 | 3,39E-05 | 0,288946 | -3,46085 | KO down vs WT |
| Dlx1 | NM_010053 | 0,000135526 | 0,000135526 | 0,310889 | -3,21658 | KO down vs WT |
| Irs4 | NM_010572 | 0,00518773 | 0,00518773 | 0,360268 | -2,77571 | KO down vs WT |
| Si | NM_021882 | 0,00209707 | 0,00209707 | 0,40525 | -2,46761 | KO down vs WT |
| Gstm6 | NM_008184 | 0,00121203 | 0,00121203 | 0,410105 | -2,4384 | KO down vs WT |
| Otp | NM_011021 | 0,00155629 | 0,00155629 | 0,411406 | -2,43069 | KO down vs WT |
| Rax | NM_013833 | 5,30E-05 | 5,30E-05 | 0,414606 | -2,41193 | KO down vs WT |
| Dlx2 | NM_010054 | 0,000300083 | 0,000300083 | 0,440216 | -2,27161 | KO down vs WT |
| Sp9 | NM_001005343 | 2,90E-06 | 2,90E-06 | 0,44246 | -2,26009 | KO down vs WT |
| Dlx5 | NM_010056 | 0,00027362 | 0,00027362 | 0,456074 | -2,19263 | KO down vs WT |
| Foxb2 | NM_008023 | 3,15E-08 | 3,15E-08 | 0,457636 | -2,18514 | KO down vs WT |
| Olig1 | NM_016968 | 4,55E-05 | 4,55E-05 | 0,466803 | -2,14223 | KO down vs WT |
| Tbx3 | NM_011535 | 0,000500565 | 0,000500565 | 0,467307 | -2,13992 | KO down vs WT |
| Arx | NM_007492 | 0,000207497 | 0,000207497 | 0,478057 | -2,0918 | KO down vs WT |
| Six3 | NM_011381 | 1,25E-05 | 1,25E-05 | 0,480345 | -2,08184 | KO down vs WT |
| Olig3 | NM_053008 | 0,00560315 | 0,00560315 | 0,490271 | -2,03969 | KO down vs WT |
| Olig2 | NM_016967 | 0,000690954 | 0,000690954 | 0,494689 | -2,02147 | KO down vs WT |
| Gsx2 | NM_133256 | 0,000545945 | 0,000545945 | 0,510516 | -1,9588 | KO down vs WT |
| Nol4 | NM_199024 | 1,11E-05 | 1,11E-05 | 0,511139 | -1,95642 | KO down vs WT |
| C130060K24Rik | NM_175524 | 7,44E-06 | 7,44E-06 | 0,512853 | -1,94988 | KO down vs WT |
| Gjd2 | NM_010290 | 0,000346777 | 0,000346777 | 0,516213 | -1,93719 | KO down vs WT |
| Gad2 | NM_008078 | 0,00274626 | 0,00274626 | 0,525892 | -1,90153 | KO down vs WT |
| Fezf1 | BC119565 | 0,00349126 | 0,00349126 | 0,532943 | -1,87637 | KO down vs WT |
| Cdh20 | NM_011800 | 8,68E-05 | 8,68E-05 | 0,548352 | -1,82364 | KO down vs WT |
| Six3os1 | NR_015385 | 8,30E-05 | 8,30E-05 | 0,551938 | -1,8118 | KO down vs WT |
| Nrxn3 | NM_172544 | 0,00343165 | 0,00343165 | 0,567385 | -1,76247 | KO down vs WT |
| Vax1 | NM_009501 | 0,00049298 | 0,00049298 | 0,57439 | -1,74098 | KO down vs WT |
| Barhl1 | NM_019446 | 1,02E-05 | 1,02E-05 | 0,57836 | -1,72903 | KO down vs WT |
| Dok6 | NM_001039173 | 5,10E-10 | 5,10E-10 | 0,578656 | -1,72814 | KO down vs WT |

| | | | | | | |
|---|---|---|---|---|---|---|
| Nol4 | NM_001161483 | 1,36E-05 | 1,36E-05 | 0,578974 | -1,72719 | KO down vs WT |
| Magel2 | BC054763 | 2,49E-07 | 2,49E-07 | 0,581115 | -1,72083 | KO down vs WT |
| Ankrd43 | NM_183173 | 0,000448456 | 0,000448456 | 0,58657 | -1,70483 | KO down vs WT |
| 6430601O08Rik | AK032580 | 0,00111031 | 0,00111031 | 0,594563 | -1,68191 | KO down vs WT |
| Lmo1 | NM_057173 | 1,36E-05 | 1,36E-05 | 0,59881 | -1,66998 | KO down vs WT |
| Rian | AF357355 | 5,65E-06 | 5,65E-06 | 0,599159 | -1,66901 | KO down vs WT |
| Nkx2-4 | NM_023504 | 8,51E-06 | 8,51E-06 | 0,608031 | -1,64465 | KO down vs WT |
| 1700101E01Rik | BC147760 | 2,53E-05 | 2,53E-05 | 0,608224 | -1,64413 | KO down vs WT |
| 4930506M07Rik | NM_001114312 | 0,000979848 | 0,000979848 | 0,610563 | -1,63783 | KO down vs WT |
| Sox1 | NM_009233 | 0,00250404 | 0,00250404 | 0,61141 | -1,63556 | KO down vs WT |
| Pdcd10 | NM_019745 | 1,15E-06 | 1,15E-06 | 0,616068 | -1,6232 | KO down vs WT |
| Rtl1 | NM_184109 | 2,95E-06 | 2,95E-06 | 0,618141 | -1,61776 | KO down vs WT |
| Nol4 | NM_199024 | 2,17E-06 | 2,17E-06 | 0,627515 | -1,59359 | KO down vs WT |
| Tbc1d30 | NM_029057 | 0,000390939 | 0,000390939 | 0,62896 | -1,58993 | KO down vs WT |
| Nr0b1 | NM_007430 | 0,00111735 | 0,00111735 | 0,63043 | -1,58622 | KO down vs WT |
| Rtl1 | NM_184109 | 5,30E-06 | 5,30E-06 | 0,635429 | -1,57374 | KO down vs WT |
| Gm9990 | ENSMUST00000070021 | 0,00196533 | 0,00196533 | 0,636752 | -1,57047 | KO down vs WT |
| 1100001E04Rik | AB073967 | 0,00056358 | 0,00056358 | 0,637139 | -1,56952 | KO down vs WT |
| Nptx1 | NM_008730 | 3,01E-05 | 3,01E-05 | 0,64667 | -1,54638 | KO down vs WT |
| Lect1 | NM_010701 | 3,30E-05 | 3,30E-05 | 0,647533 | -1,54432 | KO down vs WT |
| Pcdh9 | NM_001081377 | 0,000694503 | 0,000694503 | 0,648087 | -1,543 | KO down vs WT |
| Rtl1 | NM_184109 | 2,63E-05 | 2,63E-05 | 0,649123 | -1,54054 | KO down vs WT |
| 2310001H12Rik | ENSMUST00000089906 | 0,000100622 | 0,000100622 | 0,649417 | -1,53984 | KO down vs WT |
| Ntm | NM_172290 | 9,09E-07 | 9,09E-07 | 0,65078 | -1,53662 | KO down vs WT |
| A430033K04Rik | NM_183025 | 0,000166488 | 0,000166488 | 0,652075 | -1,53357 | KO down vs WT |
| Meg3 | NR_003633 | 1,61E-05 | 1,61E-05 | 0,656648 | -1,52289 | KO down vs WT |
| Dlx6 | NM_010057 | 0,000984513 | 0,000984513 | 0,658436 | -1,51875 | KO down vs WT |
| Lhx3 | NM_001039653 | 0,0051356 | 0,0051356 | 0,661176 | -1,51246 | KO down vs WT |
| Lonrf2 | NM_001029878 | 3,60E-05 | 3,60E-05 | 0,662943 | -1,50843 | KO down vs WT |
| 2310001H12Rik | ENSMUST00000112540 | 4,70E-06 | 4,70E-06 | 0,663584 | -1,50697 | KO down vs WT |

| | | | | | | |
|---|---|---|---|---|---|---|
| Lypd1 | NM_145100 | 0,000134161 | 0,000134161 | 0,663984 | -1,50606 | KO down vs WT |
| Dlx1as | NR_002854 | 0,00476894 | 0,00476894 | 0,664813 | -1,50418 | KO down vs WT |
| Wnt5a | NM_009524 | 0,000379905 | 0,000379905 | 1,50904 | 1,50904 | KO up vs WT |
| Olfr1418 | NM_001011524 | 9,31E-05 | 9,31E-05 | 1,51121 | 1,51121 | KO up vs WT |
| Esrp1 | NM_194055 | 0,00254113 | 0,00254113 | 1,51266 | 1,51266 | KO up vs WT |
| Myh7 | NM_080728 | 2,56E-08 | 2,56E-08 | 1,51483 | 1,51483 | KO up vs WT |
| BC061212 | BC061212 | 4,46E-05 | 4,46E-05 | 1,51574 | 1,51574 | KO up vs WT |
| Rabl5 | NM_026073 | 8,35E-07 | 8,35E-07 | 1,51648 | 1,51648 | KO up vs WT |
| Itih5 | NM_172471 | 1,25E-05 | 1,25E-05 | 1,52856 | 1,52856 | KO up vs WT |
| 5330426P16Rik | ENSMUST00000089395 | 0,00269257 | 0,00269257 | 1,52926 | 1,52926 | KO up vs WT |
| Tm4sf1 | NM_008536 | 0,000315704 | 0,000315704 | 1,52978 | 1,52978 | KO up vs WT |
| Gm8020 | ENSMUST00000095981 | 2,98E-05 | 2,98E-05 | 1,53143 | 1,53143 | KO up vs WT |
| Gm13235 | ENSMUST00000105736 | 5,04E-05 | 5,04E-05 | 1,5324 | 1,5324 | KO up vs WT |
| Gm13225 | XM_899253 | 5,04E-05 | 5,04E-05 | 1,5324 | 1,5324 | KO up vs WT |
| Foxe1 | NM_183298 | 0,000135823 | 0,000135823 | 1,53571 | 1,53571 | KO up vs WT |
| Snord115 | AF357427 | 1,90E-05 | 1,90E-05 | 1,54064 | 1,54064 | KO up vs WT |
| Snord115 | AF357427 | 1,90E-05 | 1,90E-05 | 1,54064 | 1,54064 | KO up vs WT |
| V1rd21 | NM_207547 | 8,98E-05 | 8,98E-05 | 1,54074 | 1,54074 | KO up vs WT |
| Prnd | NM_023043 | 0,000309864 | 0,000309864 | 1,54465 | 1,54465 | KO up vs WT |
| 1110032E23Rik | NM_133187 | 4,70E-05 | 4,70E-05 | 1,54814 | 1,54814 | KO up vs WT |
| Krtap4-1 | NM_001048196 | 6,93E-06 | 6,93E-06 | 1,56086 | 1,56086 | KO up vs WT |
| LOC100046559 | XR_031095 | 0,000112218 | 0,000112218 | 1,56678 | 1,56678 | KO up vs WT |
| Folr1 | NM_008034 | 0,000207585 | 0,000207585 | 1,57398 | 1,57398 | KO up vs WT |
| Slit1 | NM_015748 | 0,000190471 | 0,000190471 | 1,58333 | 1,58333 | KO up vs WT |
| Trdn | NM_029726 | 5,82E-05 | 5,82E-05 | 1,59346 | 1,59346 | KO up vs WT |
| Znhit1 | NM_027318 | 1,65E-06 | 1,65E-06 | 1,59492 | 1,59492 | KO up vs WT |
| Plod3 | NM_011962 | 1,66E-06 | 1,66E-06 | 1,61643 | 1,61643 | KO up vs WT |
| Adra1d | NM_013460 | 9,29E-05 | 9,29E-05 | 1,64217 | 1,64217 | KO up vs WT |
| Gm8304 | XM_001477007 | 4,54E-06 | 4,54E-06 | 1,65072 | 1,65072 | KO up vs WT |
| Foxl1 | NM_008024 | 0,00010926 | 0,00010926 | 1,67307 | 1,67307 | KO up vs WT |
| Cldn1 | NM_016674 | 0,00289192 | 0,00289192 | 1,68165 | 1,68165 | KO up vs WT |
| Ifitm3 | NM_025378 | 1,16E-05 | 1,16E-05 | 1,68962 | 1,68962 | KO up vs WT |
| Gja5 | NM_008121 | 4,26E-05 | 4,26E-05 | 1,7794 | 1,7794 | KO up vs WT |
| Emid2 | NM_024474 | 7,84E-05 | 7,84E-05 | 1,82318 | 1,82318 | KO up vs WT |
| Ferd3l | NM_033522 | 0,000171068 | 0,000171068 | 1,89603 | 1,89603 | KO up vs WT |
| Vgll2 | NM_153786 | 0,000193364 | 0,000193364 | 1,89767 | 1,89767 | KO up vs WT |
| Lrtm1 | NM_176920 | 0,000635917 | 0,000635917 | 1,95062 | 1,95062 | KO up vs WT |
| Corin | NM_016869 | 0,000136501 | 0,000136501 | 1,96657 | 1,96657 | KO up vs WT |
| Lgi1 | NM_020278 | 1,20E-05 | 1,20E-05 | 2,05838 | 2,05838 | KO up vs WT |
| Gal | NM_010253 | 4,83E-09 | 4,83E-09 | 2,41434 | 2,41434 | KO up vs WT |
| C1ql4 | NM_001024702 | 7,86E-06 | 7,86E-06 | 2,57204 | 2,57204 | KO up vs WT |
| Tmem184a | NM_001161548 | 4,99E-06 | 4,99E-06 | 2,612 | 2,612 | KO up vs WT |
| LOC677548 | XR_034974 | 0,0058246 | 0,0058246 | 2,96532 | 2,96532 | KO up vs WT |
| Gm9078 | XR_031413 | 6,41E-05 | 6,41E-05 | 4,17381 | 4,17381 | KO up vs WT |
