# Supplementary material for: Lhx5 controls mamillary differentiation in the developing hypothalamus of the mouse
Source: Front Neuroanat. 2015 Aug 14;9:113. doi: 10.3389/fnana.2015.00113 (PMC4536661; doi:10.3389/fnana.2015.00113)
Supplement: Supplementary Figure 1 — Medio-lateral series of sagittal sections through the brain of mouse embryos (E12.5), genotypes as indicated at the top. For each genotype, the left column shows in situ hybridization for Lhx5 and the right column shows labeling of an adjacent section with anti-LHX1/5 antibody (at higher magnification). The arrows indicate the mamillary body primordium. Scale bars left column (ISH), 500 ìm; right column (antibodies), 100 μm. [file Image1.PDF]

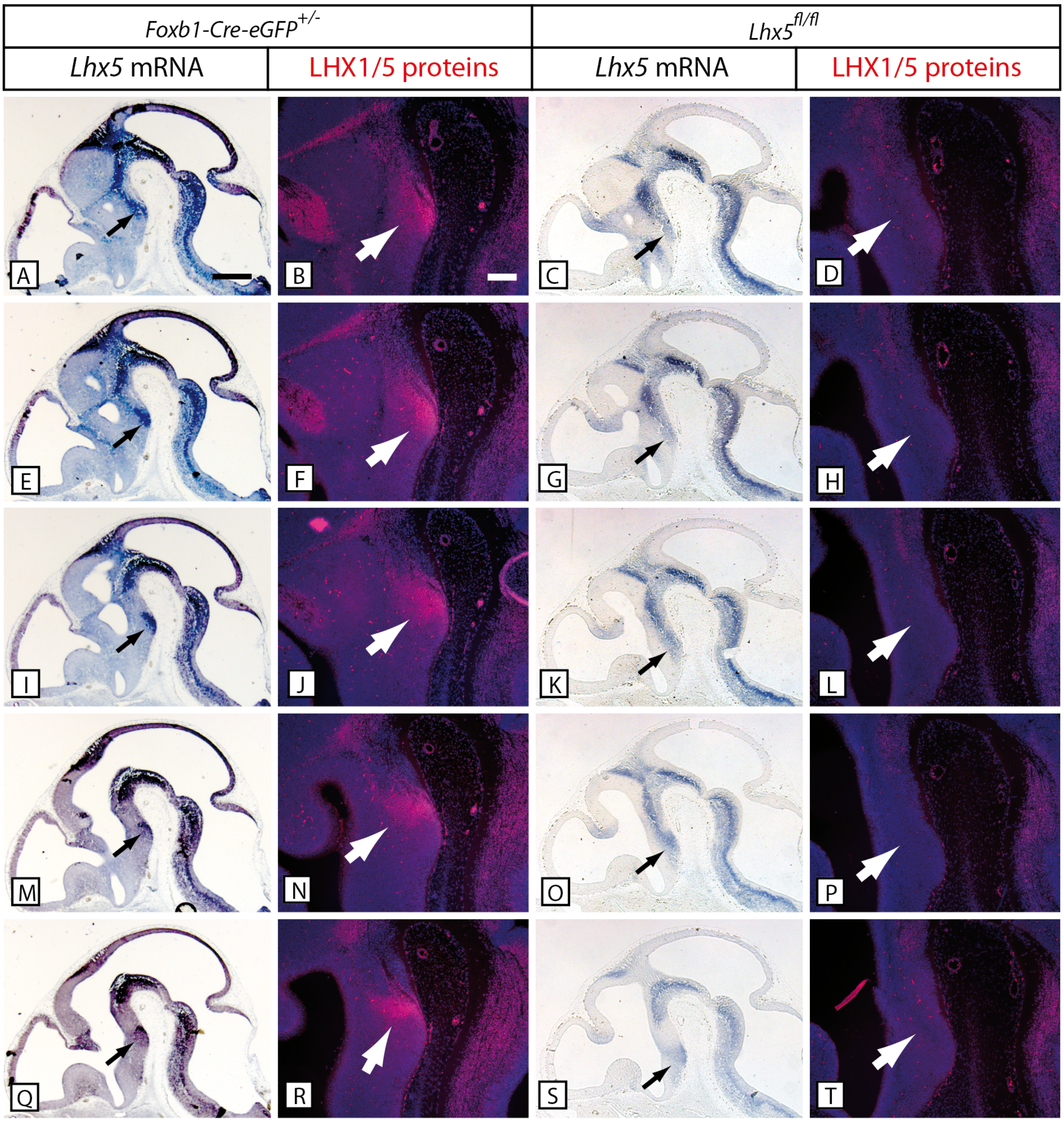

Medio-lateral series of sagittal sections through the brain of mouse embryos (E12.5), genotypes as indicated at the top. For each genotype, the left column shows in situ hybridization for Lhx5 and the right column shows labeling of an adjacent section with anti-LHX1/5 antibody (at higher magnification). The arrows indicate the mammillary body primordium. Scale bars left column (ISH), 500 µm; right column (antibodies), 100 µm.

Heide et al.

Suppl. Fig. 1
