# Supplementary figures and images for: Lhx5 controls mamillary differentiation in the developing hypothalamus of the mouse
Source: Front Neuroanat. 2015 Aug 14;9:113. doi: 10.3389/fnana.2015.00113 (PMC4536661; doi:10.3389/fnana.2015.00113)

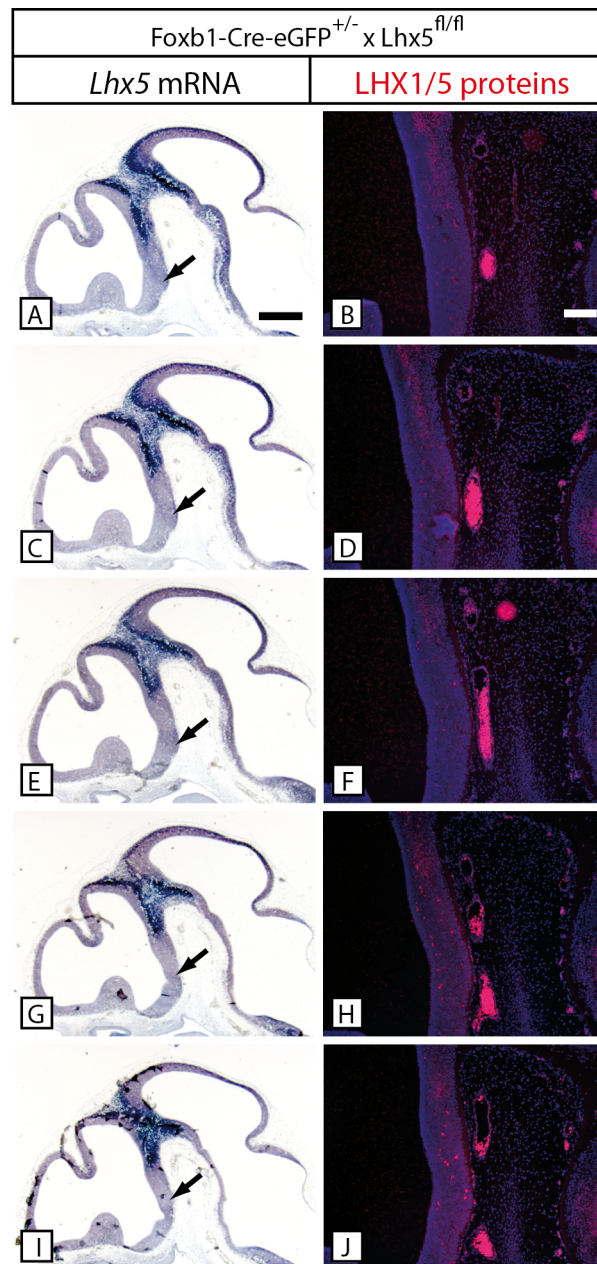

Same as Suppl. Fig. 1, for the Foxb1-Cre-eGFP<sup>+/-</sup> x Lhx5<sup>fl/fl</sup> genotype.

Heide et al.  
Suppl. Fig. 2

Supplement: Supplementary Figure 2 — Same as Supplementary Figure 1, for the Foxb1-Cre-eGFP+/− x Lhx5fl/fl genotype. [file Image2.PDF]
