# Supplementary material for: Lhx5 controls mamillary differentiation in the developing hypothalamus of the mouse
Source: Front Neuroanat. 2015 Aug 14;9:113. doi: 10.3389/fnana.2015.00113 (PMC4536661; doi:10.3389/fnana.2015.00113)
Supplement: Supplementary Figure 3 — Expression pattern of qPCR-validated candidates on Lhx5fl/+ and Lhx5fl/fl mutant embryos In situ hybridization of qPCR-validated microarray candidates (as indicated) on sagittal sections of E12.5 Lhx5fl/+ and Lhx5fl/fl mutant embryos. Insets show MBO under higher magnification. Black arrows indicate the position of the MBO; arrows after the gene name indicate downregulation (red arrow) or upregulation (blue arrow) in microarrays and qPCR. Scale bar (in A): 500 μm. [file Image3.PDF]

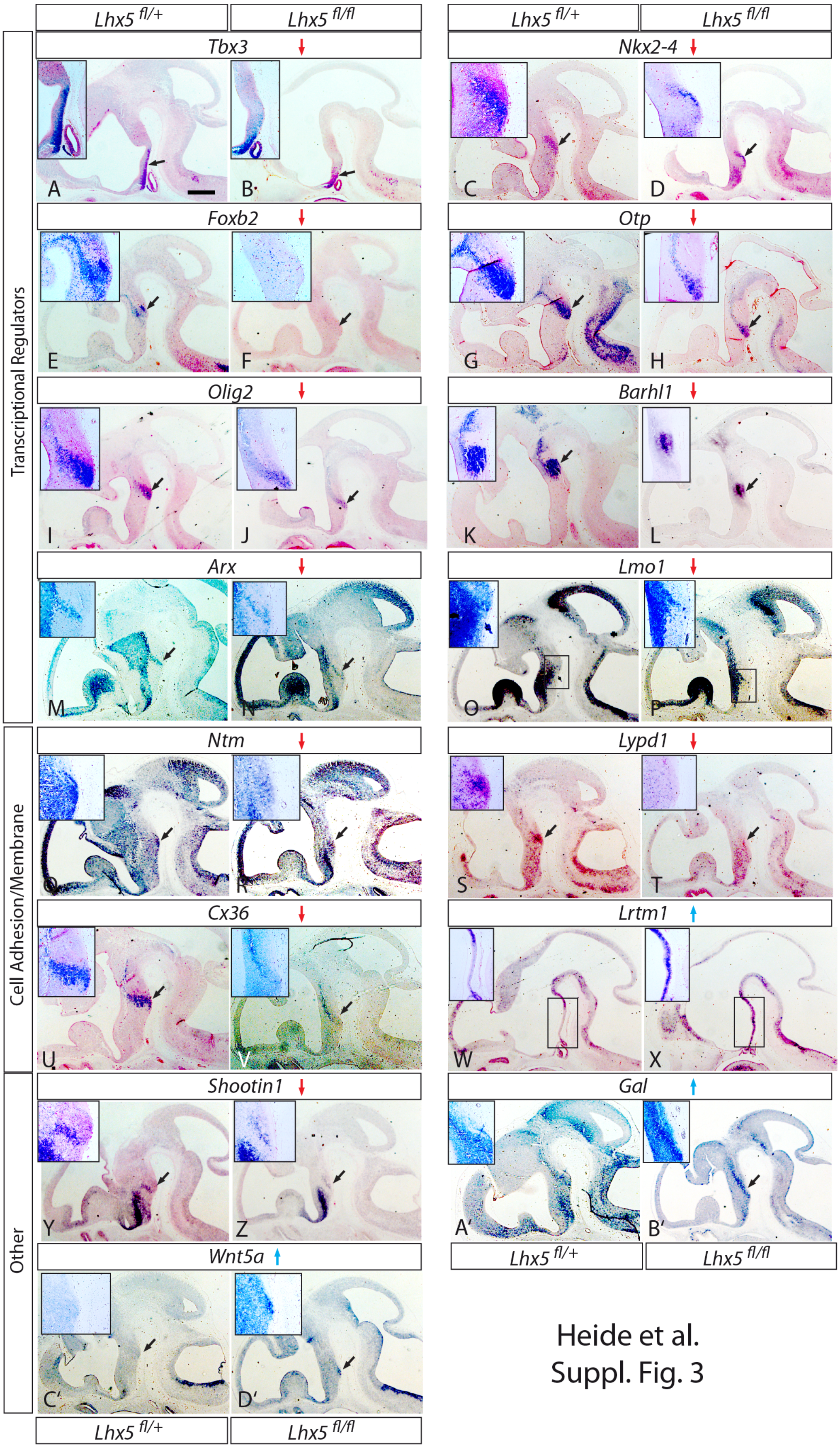

Heide et al.
Suppl. Fig. 3

Expression pattern of qPCR-validated candidates on Lhx5fl/+ and Lhx5fl/fl mutant embryos

In situ hybridization of qPCR-validated microarray candidates (as indicated) on sagittal sections of E12.5 Lhx5fl/+ and Lhx5fl/fl mutant embryos. Insets show MBO under higher magnification. Black arrows indicate the position of the MBO; arrows after the gene name indicate downregulation (red arrow) or upregulation (blue arrow) in microarrays and qPCR.
Scale bar (in A): 500 µm.
